# Colors of the Sublunar

**DOI:** 10.1177/2041669517733484

**Published:** 2017-09-29

**Authors:** Jan Koenderink, Andrea van Doorn

**Affiliations:** Justus Liebig Universität Giessen, Germany; University of Leuven (KU Leuven), Belgium; 8125 Utrecht University, the Netherlands; Justus Liebig Universität Giessen, Germany; 8125 Utrecht University, the Netherlands

**Keywords:** natural colors, ecological optics, opponent channels, spectral correlation

## Abstract

Generic red, green, and blue images can be regarded as data sources of coarse (three bins) local spectra, typical data volumes are 10^4^ to 10^7^ spectra. Image data bases often yield hundreds or thousands of images, yielding data sources of 10^9^ to 10^10^ spectra. There is usually no calibration, and there often are various nonlinear image transformations involved. However, we argue that sheer numbers make up for such ambiguity. We propose a model of spectral data mining that applies to the sublunar realm, spectra due to the scattering of daylight by objects from the generic terrestrial environment. The model involves colorimetry and ecological physics. Whereas the colorimetry is readily dealt with, one needs to handle the ecological physics with heuristic methods. The results suggest evolutionary causes of the human visual system. We also suggest effective methods to generate red, green, and blue color gamuts for various terrains.

## Motivation

We consider the colors of essentially the sublunary sphere of Aristotelian physics (itself derived from Greek astronomy). The sublunar region comprises the four classical elements (earth, air, fire, and water), the part of the cosmos where physics rules, the realm of changing nature. Nowadays, we might say “the natural environment.”

Digital photographs capture spectral information in a format that is closely related to the human visual system. This implies that the red, green, and blue (RGB) channels roughly contain counts of low-energy, medium-energy, and high-energy photons within the narrow visual range (about 1.8 to 3.4 eV). Enormous numbers of photographs from around the globe, each containing millions of spectral samples are readily available over the Internet. It would be a shame if such spectral data could not be mined and put to good use. However, there are numerous hurdles to be taken in order for this to be possible. We consider how to overcome some of them.

**Figure 1.**
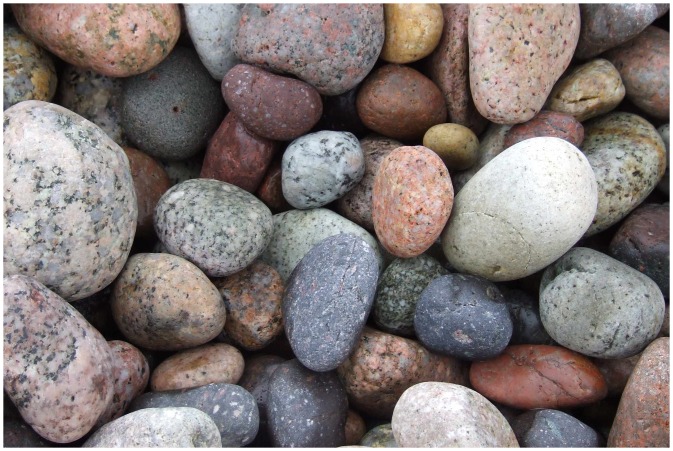
Pebbles image.

In order to motivate our methods, we start with a cursory look at the modest data source shown in Figure 1. The data volume is about $5\times 10^{6}$ samples. The average RGB color is {0.50,0.46,0.45}, which seems right from the perspective of optimal channel capacity.^1^ However, this optimistic guess is immediately shown to be wrong from a cursory glance at the covariance matrix, which is

(1) $$\begin{array}{c} C_{\mathrm{RGB}}=(\begin{array}{ccc} 97 & 94 & 92\\ 94 & 97 & 97\\ 92 & 97 & 100 \end{array}) \end{array}$$

where we have scaled the largest entry to 100. Clearly, the RGB color channels are *highly correlated*. As shown in following sections, this is entirely typical of photographs from the sublunar realm. In this case, the normalized eigenvalues are $\left\{1,2.3\times 10^{-2},2.3\times 10^{-3}\right\}$, and thus, the dominant dimension carries 40 times the power of the other two combined. The third dimension carries only a 10th of the power of the second one.

These correlations must be due to the width of the autocorrelation function of the radiant power spectra of the natural (sublunar) environment. We consider this in some detail in this article. The RGB color channel correlations have immediate consequences that are important. Here we illustrate some of these, continuing our discussion of the pebbles image.

**Figure 2.**
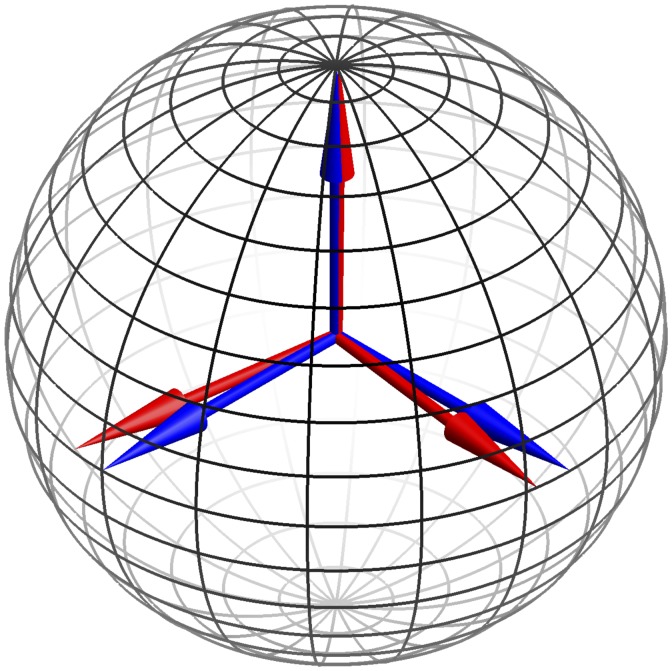
The blue vectors are $\{1,0,-1\}/\sqrt{2},\{-1,2,-1\}/\sqrt{6}$ and $\{1,1,1\}/\sqrt{3}$ and the red vectors are the eigenvectors of the pebbles image. The pole is the $\{1,1,1\}/\sqrt{3}$ direction. Note that the pebbels eigenvectors are really close to the fiducial “opponent system.”

The eigenvectors are very close to the normalized versions of {1, 1, 1}, {1,0,-1}, and {-1,2,-1}, as shown in Figure 2. Such an “opponent” basis effectively decorrelates the RGB channels. The opponent channels are white–black, red–blue and green–purple.^2^ They have an obvious interpretation in terms of physics, as discussed later.

The fact that “opponent channels” serve to decorrelate color-related signals, such as the RGB, has been known for a long time. However, this insight came from analysis of the color matching functions (Buchsbaum & Gottschalk, 1983), that is to say, the structure of the human visual system. Here we have a quite different perspective; the correlations between the RGB channels are correlations between subregions of the radiant power spectra of the sublunar realm. We do not consider the human color system, but rather the ecological optics, that is, environmental physics. This does not address the vision of any specific species. Of course, we will come back to human color vision in this article, but only by way of a detour: No doubt, human color vision evolutionary adapted to the environmental physics.

Two facts are important here. First, a default prior yields very different results. Second, as we will show later, just about all photographs deriving from the sublunar domain have essentially the same structure as that of the arbitrarily picked pebbles example (Figure 1). Why is that? This appears to be a key question from an evolutionary perspective.

The first fact results immediately from elementary probability calculus. Suppose the RGB channels are mutually independent and uniformly distributed on the interval [0,1]. This appears to be a rational default assumption that also happens to optimize the channel capacity. Then the normalized covariance matrix will be the unit matrix and the eigenvectors (except from being mutually orthogonal) essentially unconstrained. All dimensions will carry an equal share of the power. But this apparently reasonable “default assumption” is totally in the wrong ballpark.

The second fact is less easy to understand. It evidently involves the ecological physics of the sublunar realm. Accounting for this observation is a hard problem that can only be approached in a rather roundabout and approximate manner. It is dependent on the meaning of “ecological,” which not only involves the physics of the environment but also the structure of the human visual system.

In this article, we propose a model of spectral data mining that applies to the sublunar realm, involving spectra that are mainly due to the scattering of daylight by objects from the generic terrestrial environment. The model necessarily involves both colorimetry and ecological physics. The colorimetry is readily dealt with using standard tools. Because of the huge variety and complexity of the sublunar, the ecological physics has to be approached through heuristic, approximate methods of great generality.

The results yield handles on the evolutionary causes of the structure of the human visual system.

The methods described here also yield effective methods to generate RGB color gamuts for various terrains, something that might find a variety of applications.

## Psychophysical, Physiological, and Physical Backgrounds

Although we consider these backgrounds separately, they are evidently closely connected, because humans have been shaped by evolution to match their generic Umwelts.^3^ Because we are not considering visual awareness, but only discriminability, the visual part is readily dealt with using well known and standardized colorimetry. The physical part is far more involved.

### Psychophysical and Physiological Background

The human observer samples a linear projection of the radiant power spectra available at the eyes. The complement of the projection’s null-space is three dimensional for the generic human observer. The null-space of the generic projection is well known, it was established empirically in the 19th century by Maxwell and Helmholtz (Koenderink, 2010a).^4^ Nowadays, a projection matrix is available on the Internet. There is no natural basis for “color space,” that is the complement of the null-space, nor is there a natural metric.

**Figure 3.**
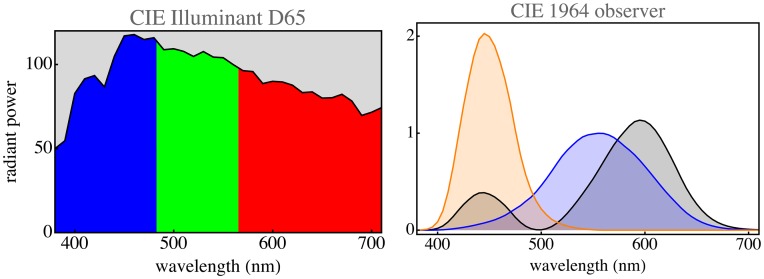
At left, the CIE illuminant D65 (average daylight). The colors show the spectral bins for the cut-loci 483 nm and 565 nm. At right, the color matching functions of the CIE 1964 supplementary standard colorimetric observer. The tables may be downloaded from the CIE site, the cut-loci can immediately be computed from them.

We consider a highly simplified model of the sublunar realm in which the radiant spectra are spectrally selectively attenuated versions of the daylight spectrum. This implements “object colors.” For simplicity, we use a standard daylight spectrum available for download on the Internet (www.cie.co.at/index.php/LEFTMENUE/DOWNLOADS; see Figure 3).

The colors of such attenuated daylight spectra fill a finite region in color space. Because the daylight spectrum defines an infinitely dimensional cuboid in the space of spectra, this region is a convex, centrally symmetric volume in color space.^5^ Its structure has been described by Schrödinger (1920). Colors on the boundary of this “color solid” are proper “parts of daylight” in the sense that their spectra are characteristic functions of connected spectral ranges or complements thereof.

**Figure 4.**
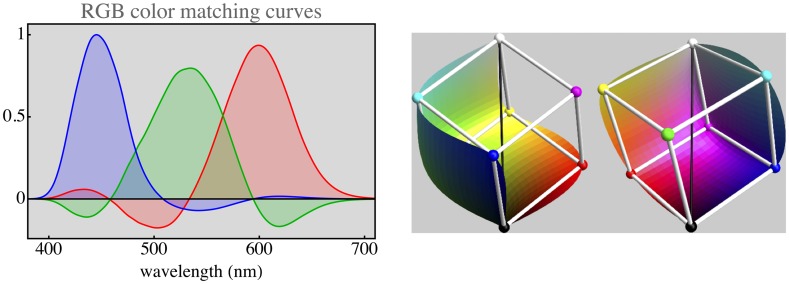
At left, the color matching functions for the parts of daylight RGB colors. At right two (mutually symmetric) halves of the surface of the Schrödinger color solid. The skeleton cube is the parallelepided spanned by the red, green, and blue parts of daylight. This is a straight calculation from the CIE tables. The RGB cube snugly fits the color solid, in practice the overwhelming majority of object colors lies in the cube. This is the theoretically optimal representation of RGB colors.

This can be used to find the nature of spectral sampling by the human visual system. Split the spectrum into three parts by way of two cuts. Place the cuts thus that the resulting RGB space claims the largest possible volume fraction of the full Schrödinger color solid. This is a well-defined optimization problem because volume ratios are invariant against arbitrary colorimetric transformations. One finds (numerically, using the CIE color matching functions shown in Figure 3 right) that there is a unique solution, and the cuts should be at wavelengths of 482.65 nm and 565.43 nm (Figure 3 left). This yields a unique RGB basis for color space. The convex hull of the basis vectors is the parallelepided of largest volume that can be inscribed in the color solid, making it the optimum RGB basis (Figure 4 right). The corresponding color matching functions (Figure 4 left) are predominantly nonnegative and are mutually only weakly correlated.^6^

Phenomenologically, the resulting parts look red, green and blue to generic observers, whereas unions of two parts look yellow, turquoise, and purple and the union of all three parts looks white.^7^ Thus, one has a true RGB representation, exactly what display manufacturers aim for. If a display deviates significantly from this optimum, it is unlikely to attract customers. The reason is simply that the physiology dictates it.

Of course, there is no necessity for display manufacturers to produce “parts of daylight” as such. For display purposes, they are already in good shape when they get the *colors*—not necessarily the *spectra*—right. Thus, one might even use (quasi-)monochromatic sources. In practice, the spectra will often derive from the electronic structure of rare earth elements, from various organic molecules and so forth and often be rather rough. Nevertheless, the gamuts of current display units approximate that of the parts of daylight.

The same does not apply to the sensors. Ideal sensors would implement the human projection (Figure 4 left). The parts of daylight would be a good choice that is approximately physically possible because the sensor sensitivities should be nonnegative throughout most of the spectrum. Of course, such an ideal cannot be achieved. In practice, one makes do with coarse approximations. This typically involves a mosaic of absorption filters in front of the CCD or CMOS photosensitive chip. Fortunately, this tends to work out fine because almost all spectra of interest are not highly articulated. This is a topic to be discussed in the next section.

This suggests that human physiology effectively implements hyperspectral imaging with three bins per pixel, the bins being (0,483nm),(483nm,565nm) and (565nm,∞), where—in practice—“0” is really somewhat like 380 nm and “∞” somewhat like 700 nm. The effective visual range subtends hardly an octave.^8^ Of course, the precise locations of the bin boundaries depend upon the daylight spectrum and the color matching functions. From a biological perspective, the key role of the daylight spectrum in setting up the RGB basis makes good ecological sense. The color matching functions are expected to be evolutionary tuned to it, indeed, various suggestions have been proposed in the literature.

This spectral description in terms of three bins is a natural RGB system, to which the camera and display industries have to comply—of course, approximately and by various heuristics. In practice, one notices that displays have largely converged, whereas there is quite a bit of variation among sensor sensitivities. That is why the “color rendering” of cameras tends to be debated in websites reviewing the latest consumer cameras. However, to the first approximation, all cameras are very similar, or they would not attract any customers at all.

This is essentially all the colorimetry needed in this article. Note that we do not refer to qualities of visual awareness, nor to just noticeable differences and so forth.

### Physical Background

#### The physics is rather more involved

In order to avoid unfortunate confusion, it is necessary to distinguish between the spectrum of radiative power (henceforth called RP spectrum) and the spectrum of the articulation of the RP spectrum (henceforth called SA spectrum). The SA spectrum is the Fourier transform of the envelope of the RP spectrum. It can be quantified in terms of cycles per octave of the RP spectrum (Koenderink, 2010a). Both the amplitude and phase of the SA spectrum are relevant.

From the perspective of physics, the visual range subtends only a narrow window of the electromagnetic radiant power spectrum (about 380–700 nm as mentioned above). This is highly relevant from an ecological perspective, for the physical causes of spectral articulations change categorically over the electromagnetic spectrum (Feynman, Leighton, & Sands, 1963–1965). Molecular rotation bands occur in the infrared spectrum, while effects of electronic transitions in atoms occur in the ultraviolet spectrum. Articulation in the visual range is largely due to processes involving chemical binding energies. Since the set of physical causes is the same over the visual range and the width of the range is only an octave, the range will be statistically uniform. For spectral articulation, the important processes may be taken as translationally (along the wavelength axis!) invariant. This implies that a spectral analysis (the SA spectrum) makes sense. The articulation can have a variety of causes, there appears to be no particular absolute dimension. Thus, the default assumption would be scale-invariant (or self-similar) spectral statistics (Chapeau–Blondeau, Chauveau, Rousseau, & Richard, 2009)

It is hard to put this to an empirical test. Estimates of the SA spectra for a small number of rather narrowly focused databases appear to confirm the notion. However, one is stuck with an annoying lack of data (Kohonen, Parkkinen, & Jaäskelaïnen, 2006). An analysis of the available data appears to conform to expectations though. Some examples can be found in Koenderink (2010a).

Perhaps surprisingly, these simple notions are already sufficient to draw some important consequences. Given that the visual range is narrow and its structure translation invariant, one expects the covariance matrix of the RGB color channels to have a structure roughly like

(2) $$\begin{array}{c} C_{\text{RGB}}\propto (\begin{array}{ccc} 1 & 1-{ɛ}_{1} & 1-{ɛ}_{2}\\ 1-{ɛ}_{1} & 1 & 1-{ɛ}_{3}\\ 1-{ɛ}_{2} & 1-{ɛ}_{3} & 1 \end{array}) \end{array}$$

where the ${ɛ}_{1,2,3}$ are positive and (typically much) smaller than 1, whereas—because covariance will be a monotonic function of spectral separation—one expects ${ɛ}_{1}\approx {ɛ}_{3}$ and ${ɛ}_{2}$ to be significantly larger than ${ɛ}_{1,3}$. This approximate form is expected because there is no reason why the color channels should be distinguished, the covariance should only depend monotonically upon spectral distance^9^ (Koenderink, 2010b). Indeed, letting the data speak (section “Let the data speak”) fully bears this out.

For simplicity, we consider the case ${ɛ}_{1}={ɛ}_{3}=ɛ,{ɛ}_{2}=2ɛ$ as an illustration. To the lowest relevant order in *ɛ* (zero or one), the eigenvectors of $C_{\text{RGB}}$ are

(3) $$\begin{array}{c} e_{1}=\frac{1}{\sqrt{3}}(\begin{array}{c} 1\\ 1\\ 1 \end{array}),e_{2}=\frac{1}{\sqrt{2}}(\begin{array}{c} 1\\ 0\\ -1 \end{array}),e_{3}=\frac{1}{\sqrt{6}}(\begin{array}{c} -1\\ 2\\ -1 \end{array}) \end{array}$$

and the corresponding eigenvalues proportional to 1, $\frac{2}{3}ɛ$, and $\frac{2}{9}ɛ$. These eigenvectors are similar to white–black, red–blue, and green–purple “opponent” channels as originally proposed by Hering (1920) on phenomenological grounds.

The first eigenvalue strongly dominates. It carries Z=9/(8ɛ) times the power of the other dimensions combined. This ratio Z is a useful characteristic number that is easy to derive from image databases, and it will be used in the section on data mining (section “Let the data speak”). It tends to be significantly larger than one (about three to thirty in practice). Note that the higher the Z, the closer the images are to being effectively monochrome. In almost all ecologically relevant cases, the first eigenvalue so strongly dominates that it will typically make sense to treat the second and third dimensions as essentially independent of the first one. These two eigenvalues are seen to be in a fixed ratio (here three).

This rough analysis is interesting in view of the significant literature on principal component analyses of collections of empirically determined spectral reflectance factors (Fairman & Brill, 2004; Tzeng & Berns, 2005).^10^ Resulting principal components are invariably similar to the eigenvectors derived above (further illustrated below), there is essentially no valid reason to go through the trouble of measuring them and there is little reason to expect differences for various collections of samples. Indeed, there are not. The minor differences reported are probably due to the necessarily (very) limited size of the samples, which is perhaps an additional reason to prefer a fixed, formal basis.

The reason for the prominence of the two (instead of three^11^) opponent-like eigenvectors is that they implement the first- and second-order derivatives of the SA spectrum (see below). Thus, these opponent channels represent the structure of the SA spectrum at a point. This also explains why they are mutually independent, it derives from the independence of derivatives of noise signals (such as the SA spectrum) of even and odd order (Longuet–Higgins, 1957).

This analysis, indeed, accounts for the major traits of the empirical data is illustrated by the simulations presented in Appendix A.

#### The physics of “object colors”

Object colors are due to radiant spectra that largely result from the scattering of radiation—here to be taken as average daylight say—by solids. Generic examples are colored papers, fabrics, human skin, soil, and rocks, … There are various processes that may play a role.

An important process is the radiative transport in layered turbid media. A well-known, approximate model is the Kubelka–Munk theory of turbid layers (Kubelka & Munk, 1931).^12^ It is an approximate treatment of the radiative transport in layered turbid media that is very successful in applications and widely used in the paint, paper, and so forth industry. We introduce it here as a heuristic aid.

The key expression of the Kubelka–Munk analysis is

(4) $$\frac{1-R_{\infty }^{2}}{2R_{\infty }}=\xi (=\frac{K}{S})$$

where $R_{\infty }$ is the reflectance of an infinitely thick layer, *K* is the specific absorption cross-section, and *S* is the specific scattering cross-section. Solving for $R_{\infty }$ yields the inverse relation

(5) $$R_{\infty }=\sqrt{1+(\xi {)}^{2}}-\xi =F(\xi )$$

The function F(ξ) maps between the nonnegative reals (0,∞) and the unit interval (0, 1).

From a global perspective, the structure of the Kubelka–Munk result is that the nonlinear part of the theory is packaged in the left side of Equation (4), whereas the right side of this equation describes fundamental physical causes—responsible for the spectral articulation—which are dominated by linear processes. We use these observations as a heuristic.

In ecological optics, one really does not have explicit theories; rather, a possibly large number of mutually different processes is likely to play some role. There is a need to capture this in a general, overall way. Here, one may take a lead from the formal structure of the Kubelka–Munk equation (though not necessarily the explicit Kubelka–Munk theory itself). That is what will be attempted here.

The scattering and absorption cross-sections are nonnegative physical quantities for which there exists no preferred absolute scale. Thus their noninformative Jeffreys’ prior distribution (Jaynes, 1968; Jeffreys, 1939, 1946) is hyperbolic, that is uniform on the logarithmic scale. Moreover, the quantities *K* and *S* are mutually uncorrelated. Thus, the parameter *ξ* (the ratio *K*/*S*) also has the hyperbolic prior.

The physical parameters combine multiplicatively, rather than additively, so a logarithmic representation is a natural one for the statistics.

A convenient way to capture this is to define a transformation Ω from the full real line ℝ (on which the “physical parameters” are uniformly distributed) to the unit interval I (the observer intensities in the RGB channels, taking values between zero and one) and back. For convenience, one may use the pair

(6) $$\Omega (x)=\frac{1}{2}(1+\tanh x)\Omega :\mathbb{R}\to \text{I}$$

and

(7) $$\Omega^{-1}(y)=\text{atanh}(2y-1)\Omega^{-1}:\text{I}\to \mathbb{R},$$

because these transformations have fast implementations on most computing platforms. This is important since they may have to be applied a hundred million times in some typical example. From a general point of view about any sigmoid shaped function, such as (1+erf(x))/2 and so forth, would serve as well.

Note that the cases $\Omega^{-1}(0)=-\infty$ and $\Omega^{-1}(1)=+\infty$ are always to be avoided for technical reasons since the boundaries of the interval tend to accumulate physically meaningless observations due to under or overexposure.

In practice, one transforms observations on the unit interval to the “physical domain” (the full real line), does some calculations, and transforms back. It is an instance of the so-called homomorphic filtering (Oppenheim, Schafer, & Stockham, 1968), where the observations and calculations take place in distinct, appropriate domains. In our case, we collect data in the observation domain and study its statistics in the physical domain; in other applications, one generates artificial data in the physical domain and studies it in the observation domain. Examples follow below. It is a way to avoid nonlinear unpleasantness cheaply.

#### The physics of the imaging process

In the case of *imaging*, one may use a formally very similar phenomenological model (Barrett & Myers, 2003).^13^ Here, the radiances in the scene are mapped on the unit interval for each of the color channels. When log radiance is mapped with the function Ω, the parameters are usually termed “exposure” (the location) and “contrast” (the width). Such a mapping is usually followed with a “gamma transformation” (Poynton, 2003), for example, $r\to (r/r_{0}{)}^{\gamma }$ with γ>0 and not to different from 1.

Although perhaps surprising at first blush, it makes intuitive sense that RGB photographs should retain the signature of the articulation of the radiative power spectral envelope, at least in some coarse fashion. If it was not the case, the images would not be acceptable to generic viewers. A formal calibration is not required, but typically one should be able to judge the distinction between red and green image details from the relative magnitudes of the RGB channels.

Of course, there are a variety of other factors that might put the value of potential “data” in jeopardy. The transformations considered above also handle the spatial nonuniformity, such as the focal plane illumination fall-off of generic cameras. The major remaining source of worry is probably transverse chromatic aberration. Fortunately, it is not too prominent (at least after correction by the in-camera firmware) in most contemporary camera models. It is unlikely to have an important effect on the statistics anyway, since it occurs at linear features, whereas the bulk statistics derives from areas.

#### A Phenomenological Ansatz

In the present application to the colors of the sublunar, the *data* are the color channels of images obtained by some familiar process (CCD or CMOS camera using RGB Bayer pattern say) and distorted for visual display (the Internet say). There are no radiometric calibrations. It is a very roundabout and most likely distorting way to observe physical parameters in the scene. Only by considering *relations between relations* one can expect to zoom in to relevant structure, absolute values cannot be expected to be informative.

Suppose the “true” radiometric signals in some specific case were {*r*, *g*, *b*}. Let the display distortion apply different magnifications $\left\{A_{r},A_{g},A_{b}\right\}$ (say) and different gamma corrections $\left\{\gamma_{r},\gamma_{g},\gamma_{b}\right\}$ (say) to the color channels, so one observes $(A_{r}r{)}^{\gamma_{r}}$ instead of *r*, and so forth. Does this have a major impact on the observed covariance structure? The question is most conveniently answered through a simulation. With *γ*’s in the range (0.5,1.5) and magnifications in the range (0.5,1.5), which is a wide range for typical “corrections,” the median correlation became 0.991, with interquartile range (0.973,0.998). Apparently, the covariance structure of the color channels easily survives maltreatments as one expects them for images retrieved from the Internet.

More generally, monotonic transformations due to a variety of physical factors are unlikely to have much effect. This is perhaps intuitively reasonable given the fact that at least rank order correlations are not sensitive to such factors at all.

**Figure 5.**
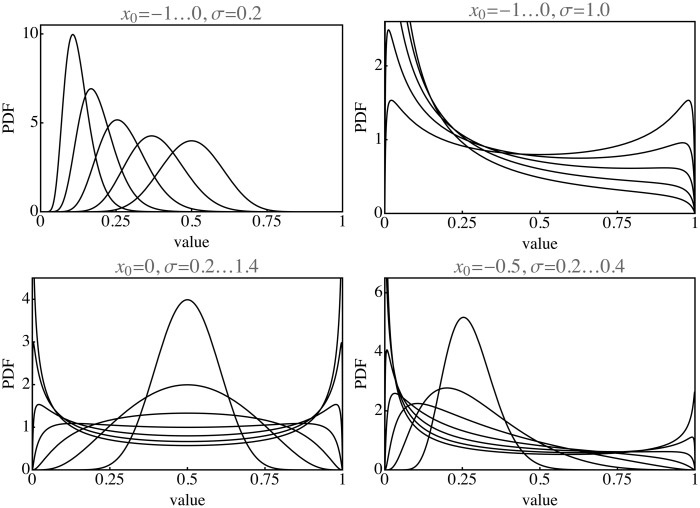
Example of histograms in the observation domain due to normal distributions of various means and variances in the physical domain. Note that these are far from normal in the observation domain.

The physics may be statistically modeled by a normal distribution on the logarithmic scale, characterized by location and width, for some physical parameter ϱ (say) in analogy to the *ξ* parameter of the Kubelka–Munk theory. A highly schematic model of the generalized physics might be a sigmoid function Ω, mapping the logϱ domain on the unit interval. This leads to reflectances whose distribution depends on two parameters. Depending on the values of the parameters, one obtains histograms that are unimodal and skewed to either zero or one, or histograms that are bimodal with peaks at zero and one (see Figure 5). This is indeed very similar to what is encountered in empirical data. Such a schematic model of the generic physics captures the essential structure. (Kubelka–Munk theory being one illustrative instance.) The two parameters have to be estimated from empirical data, for this is a purely phenomenological model.

## Let the Data Speak

Even a medium-sized image^14^ contains many pixels, for instance a 512 × 512 image contains more than a quarter million pixels (262,144 pixels). Thus, it is often possible to obtain useful statistics from a single image. On the other hand, the typical RGB image uses byte encoding, thus resolves 256^3^, that is almost 17 million bins in the RGB cube. The 512 × 512 image can at most fill 1.6% of the bins with one sample each. In order to have an average bin content of a hundred one needs more than 6,000 of such images.

Typical images today range from about 32 × 32 (“icon”, 1 kp) to 4096 × 4097 (consumer digital camera, 16Mp). For a typical field of view of $50^{\circ }$, a pixel averages over 1–2°, down to 1–0.5′. In terms of linear size, one needs to multiply with the distance, which typically ranges from arm’s length (immediate environment) to many miles (landscapes). Thus, the relevant physics might be mutually very diverse for the pixels.

As an example of single image statistics, we proceed with the image of pebbles (Figure 1). It is a medium-sized image, it measures 2736 × 1824 pixels (thus about 5 Mp). The image is JPEG compressed, thus contains numerous artifacts on the local spatial scale. The overall mean RGB pixel value is {50, 46, 45},^15^ thus somewhat skewed towards the red, but approximately a median gray, as expected.^16^ We already reported the covariance of the raw {*r*, *g*, *b*} values.

As a first operation, the RGB channels are transformed to ϱχβ, or “physical space” (using the function $\Omega^{-1}$). The normalized covariance matrix becomes

(8) $$\begin{array}{c} C_{\varrho \chi \beta }=(\begin{array}{ccc} 92 & 90 & 89\\ 90 & 95 & 96\\ 89 & 96 & 100 \end{array}) \end{array}$$

It has a very similar structure as found for the raw values (Equation (1)). What has changed are the *distributions*. The raw {*r*, *g*, *b*} values have histograms that may vary a lot, whereas the transformed values are close to being normally distributed. The transformation

(9) $$\begin{array}{c} (\begin{array}{c} \Lambda \\ \Theta \\ \Xi \end{array})=T(\begin{array}{c} \varrho \\ \chi \\ \beta \end{array}),\text{where}T=\frac{1}{12}(\begin{array}{ccc} 4 & 4 & 4\\ 6 & 0 & -6\\ -3 & 6 & -3 \end{array}) \end{array}$$

finally yields the parameters {ΛΘΞ} that will be used in the analysis of the data. These parameters are nearly decorrelated and the first one, Λ, strongly dominates. Indeed, one finds (here normalized on a maximum coefficient of 1,000)

(10) $$\begin{array}{c} C_{\Lambda \Theta \Xi }=(\begin{array}{ccc} 1000 & -25 & 6\\ -25 & 39 & -4\\ 6 & -4 & 4 \end{array}) \end{array}$$

The various covariance matrices thus have pretty much the form expected from first principles. Thus, already from a single image, the major aspects of the sublunar color gamut are apparent. Note that the scene contains mainly diffusely scattering solids, no sources or metallic reflectors and so forth.

For this image Z=24.6, as expected, much higher than unity. Since the Λ channel dominates so strongly over the ΘΞ ones, it makes sense to split the two. One uses the fraction of the variance captured by the Λ channel as one observation and the (normalized) covariance matrix for the ΘΞ plane as another. The Θ channel accounts for almost all of the remaining variance, which is entirely typical. Moreover, one has $\begin{array}{c} C_{\Theta \Xi }=(\begin{array}{cc} 100 & -10\\ -10 & 9 \end{array}) \end{array}$.

An important gain of this transformation is that the ϱχβ histograms in the “physical domain” are much closer to normal than in the bare color channel domain. The Λ histogram is close to normal too, whereas the Θ and Ξ histograms look somewhat more complicated. Indeed, typically most of the idiosyncrasy of an image tends to be found in these components.

Of course, this is just a very small sample. Because a small sample, it is perhaps in danger of being atypical. For larger databases, the idiosyncratic nature of singular images tends to be drowned in the crowd.

**Figure 6.**
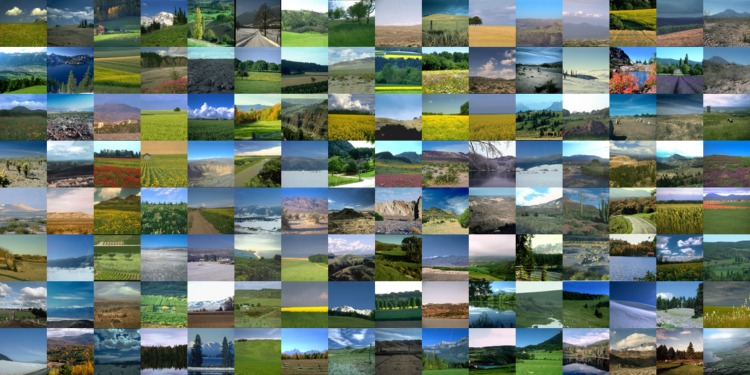
A mosaic composed of a subsample of the “open landscape” set of the MIT database.

**Figure 7.**
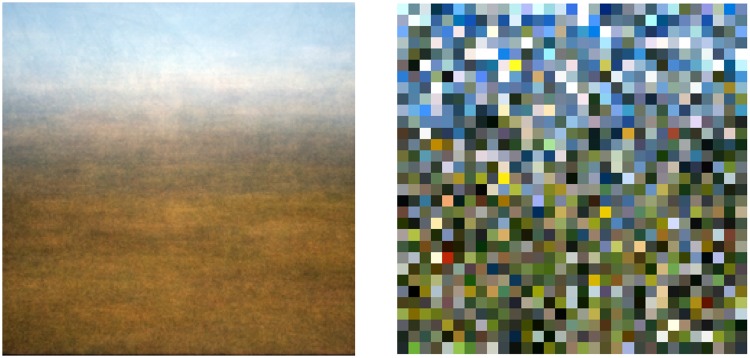
Local mean (left) and local samples (right) for the open landscape database.

More extensive statistics is available from a variety of databases in the public domain. The landscapes database from Torralba and Oliva at MIT (Torralba & Oliva, 2002) is an example (Figure 6). It is an interesting case because it also allows a distinction between what is intended as “sublunar” here and what might be termed “aerial,” or “atmospheric.” The database contains 410 “open country” images in total. All are 256 × 256 pixels, 8 bit per RGB channel. The majority has a strip of sky on top and a strip of foreground at bottom (see Figure 7 left). In the analysis, the “top” was defined as the upper 64 rows of the image pixel array and the “bottom” as the lower 64 rows of the image pixel array. Although obviously not exact, this certainly serves to split the data in a group that is predominantly sky, or atmospheric and a group that is predominantly “sublunar” in the intended sense of this article. This reduces the volume of the top and bottom sets to about 6.7 Mp samples each.

Indeed, simply averaging over all images in the database yields a “generic landscape” image that is brownish below and bluish on top. Many of the images include blue sky (Strutt, 1899). It is the kind of priming, which a landscape painter might use in preparation of a painting. The human visual system is also tuned to this type of color banding (Koenderink, van Doorn, Albertazzi, & Wagemans, 2015).

Of course, the averaging removes all local variety. The nature and extent of this variety is retained in sampled images (see Figure 7 right). Each instance of such a sampled image is different, because pixel values are randomly sampled over the whole database, the only invariant being location in the pixel plane.

The effect of the air–light (Koschmieder, 1924) is visible in the average image, both in the ground plane and in the sky. The colors of the distant ground plane and the low sky become very similar at the horizon (Middleton, 1952). Apparently, such facts of ecological physics are quite robust in the sense that they survive noncalibration and likely maltreatment of image processing. Large data speak so loudly that these problems are overcome in the statistics.

**Figure 8.**
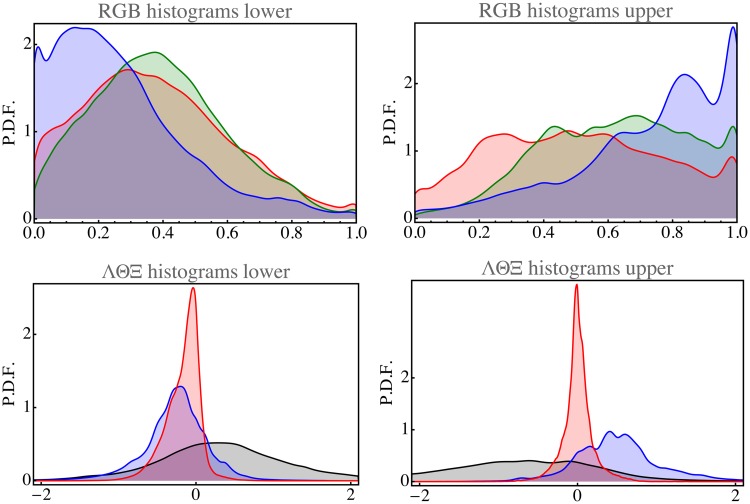
Histograms for the open landscape database. Top row for the observation domain and bottom row for the physical domain (black: Λ, blue: Θ, and red: Ξ). The left column relates to the lower (earth) part and the right column to the upper (aerial) part of the images.

The average RGB levels of the top part is {50, 63, 73}, that of the bottom part is {39, 40, 28}. Thus, the bottom part indeed looks brownish on the average, the top part bluish. This is also evident from the RGB histograms (Figure 8). Note that the histograms are far from normal, as could hardly be expected otherwise.

A transformation to physical space makes the histograms, although somewhat skew, appear much more normal. Of course, the precise form depends somewhat on the choice of the sigmoid transfer function. The {Λ,Θ,Ξ} values are nearly normally distributed (Figure 8).

The differences between the sky and earth parts of the open landscape images are well captured by the means and standard deviations of the {Λ,Θ,Ξ} parameters. One has Λ=-0.350±0.500, Θ=0.153±0.239, Ξ=0.009±0.068 for the earthy part of the images and Λ=-0.273±0.595, Θ=-0.311±0.320, Ξ=0.078±0.081 for the aerial part.

**Figure 9.**
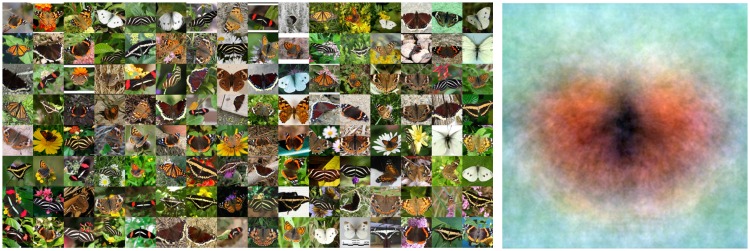
The Leeds butterflies database. At left some samples, at right the overall mean.

**Figure 10.**
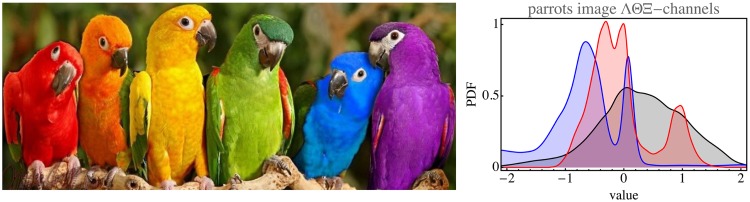
Parrots image with its histograms in the physical domain (colors as in Figure 8).

Such parcellated structure as in the open country database is quite typical for focused databases. As an example, the global mean of the Leeds butterfly database (762 images after removal of the images of pinned insects from museum collections) clearly reveals a “generic butterfly” (Figure 9). Such material is evidently unsuited to the present purpose. The same goes for images that depict various mutually very different items. An example is the parrots image (Figure 10). Not surprisingly, the ΛΘΞ histograms are far from normal here. Thus, the method of chromatic data mining as discussed here only makes sense for reasonably homogeneous images or databases.

Here, we show some examples aimed at various types of terrain, some based on fairly large, representative images, other on databases focused on particular topics. For more information on the databases, see Appendix B.

**Figure 11.**
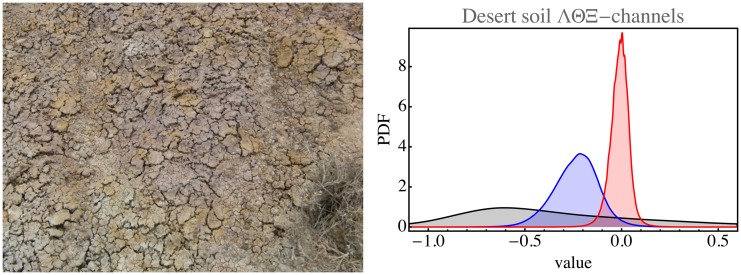
Desert soil image with its histograms in the physical domain (colors as in Figure 8).

An image like the desert soil image (Figure 11) is obviously quite homogeneous. It is a fairly large image (3264 × 2448 pixels), yielding a data volume of 8 Mp. The structure is entirely standard, with Z=8.3. The ΛΘΞ histograms are close to normal. Here, the analysis applies perfectly. The same applies to most images of landscapes selected for uniformity.

**Figure 12.**
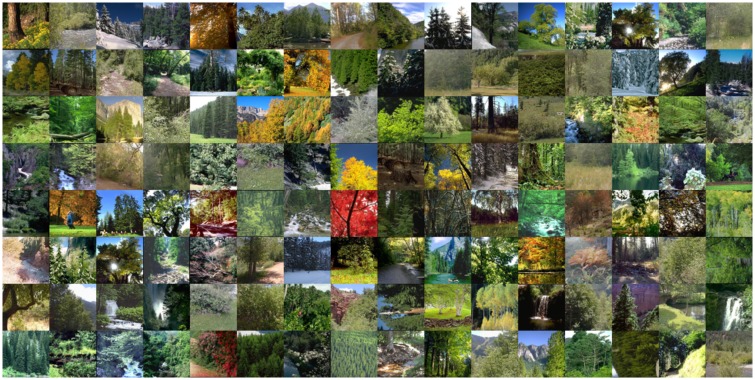
Samples from the forest data base. It is very inhomogeneous.

**Figure 13.**
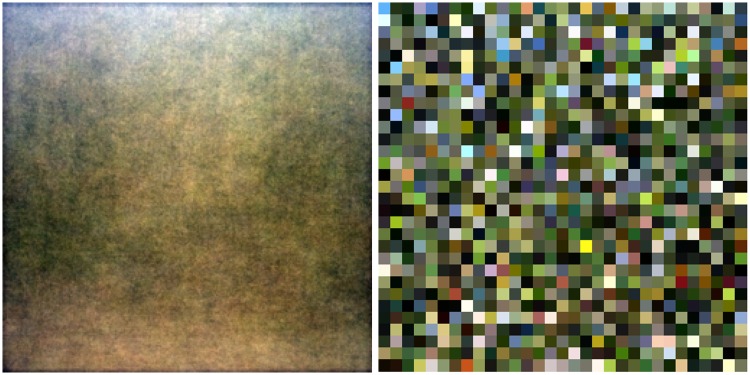
Local overall mean and local samples from the forest database.

Databases tend to be less overall uniform, though this need not be much of a problem if the fraction of “outliers” is small. As an example, consider the forest database (Figures 12 and 13). Here, two distinct types of nonuniformity occur.

*First*, there are outliers such as autumn foliage. Since these are true outliers, they are not problematic due to sheer numbers.

*Second*, there is a systematic trend for blue sky intruding on the top part. Here, large numbers do not help as can be seen from the global average. As a result the ΛΘΞ histograms appear as perturbed normal distributions. The only remedy is to cut off the top part of all images.

There are evidently detectable differences in the available databases, although the overall structure is quite invariant. This can be judged in the larger databases by sampling random subsets. One finds that the statistical estimates for samples of say a hundred images (that is still good for millions of pixels) are very stable and well determined. Since any sample from one of the databases yields pretty much the same results, the databases have a unique signature, despite their global similarity. This suggests that the description might have some merit as a descriptor of the “gist” (Oliva & Torralba, 2006)—in colorimetric respects—of a database.

**Figure 14.**
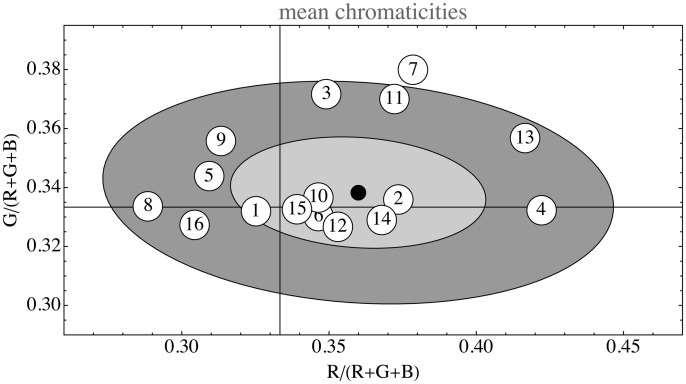
The overall RGB mean. The horizontal and vertical guidelines denote the one-third values, thus their intersection marks the point *R* = *G* = *B*. The ellipses show the one and two standard deviations boundaries. The indices refer to the list of data sources (see Appendix B). In total, this figure is based on $5\times 10^{9}$ RGB samples.

As to be expected, the images one encounters are almost invariably normalized so as to be overall medium gray with maximum contrast. The overall RGB means scatter all about the achromatic point in a chromaticity diagram (Figure 14). A measure of the monochrome contrast is the standard deviation in Λ. Empirically it varies over the range 0.65–1.38 (quartiles [0.99,1.07,1.10]). This range is very limited, no doubt due to automatic, in-camera range selections, thus essentially meaningless for ecological research.

Meaningful measures are necessarily modulo Λ. For the examples analyzed in this article, the characteristic number Z ranged from 1.8 to 23, quartiles {2.97,4.43,8.00}. Thus, all were much larger than the value expected for mutually independent, normally distributed with equal variance ϱ,χ,β channels. The number Z and the average value of Λ are mutually uncorrelated, thus Z is a meaningful number.

**Figure 15.**
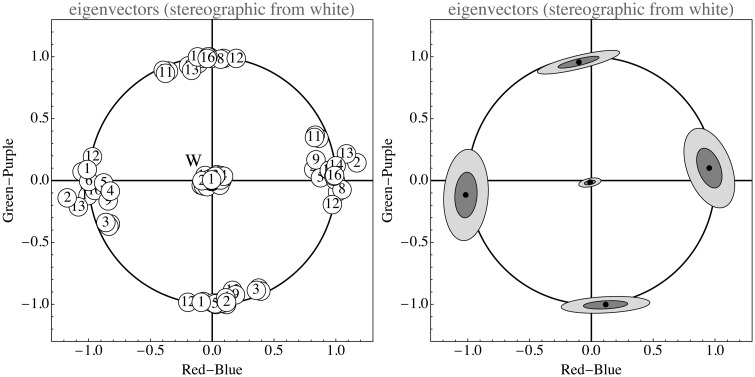
Opponent color frame. These are stereographic projections of the sphere of eigendirections from the point $\{1,1,1\}/\sqrt{3}$. The circle is the locus of orthogonal directions to $\{1,1,1\}/\sqrt{3}$. There is an obvious clustering along the “opponent directions.” The ellipses show the one and two standard deviations boundaries. The indices refer to the list of data sources (see Appendix B). In total, this figure is based on $5\times 10^{9}$ RGB samples.

As can be seen in Figure 15, the opponent channel frame indeed fits almost universally. In this figure, the eigendirections of the ΛΘΞ covariance matrix have been plotted in a stereographic projection from the white point (thus $\{1,1,1\}/\sqrt{3}$). The first eigendirection is closely centered on the origin, much as expected. The remaining two eigendirections are indeed strongly clustered and are close to the expected $\pm \{-1,0,1\}/\sqrt{2}$ (red–blue opponent) and $\pm \{-1,2,-1\}/\sqrt{6}$ (green–purple opponent). Thus, the data speak strongly in favor of Hering’s (1920) opponent system. These directions, thus, are strongly implicated by billions of spectral samples, there is no phenomenology of chromatic qualia involved.

For the data in Figures 14 and 15 (see Appendix B), we used a set of 5 large single images and 11 databases, some very large. The collection is very heterogeneous, for instance, the landscapes were not segmented into foreground and sky, the flowers and butterfly databases were used as is and so forth. It is interesting to see how the structure of all these sets is rather similar although very different from the apparently obvious default assumption (mutually independent, uniformly distributed RGB channels).

For large samples, the pixel RGB data are largely captured by four parameters, describing the level variability of the spectral articulation as described by Θ and Ξ. For smaller samples, one encounters deviations from normality in the distributions of Θ and Ξ, sometimes finding bimodality, more typically heavy tails instead of normality. The Θ and Ξ distributions capture the spectral articulation, which will naturally vary from sample to sample when the sample size is small.

The standard deviation in Θ varied over the range 0.21–0.76 (quartiles [0.35,0.47,0.55]). It is a measure of the cool–warm contrast (Benson, 2000), the variation of spectral slope.

The standard deviation in Ξ varied over the range 0.05–0.29 (quartiles [0.10,0.13,0.19]). It is a measure of the moist–dry contrast (Benson, 2000), the variation of spectral curvature.

**Figure 16.**
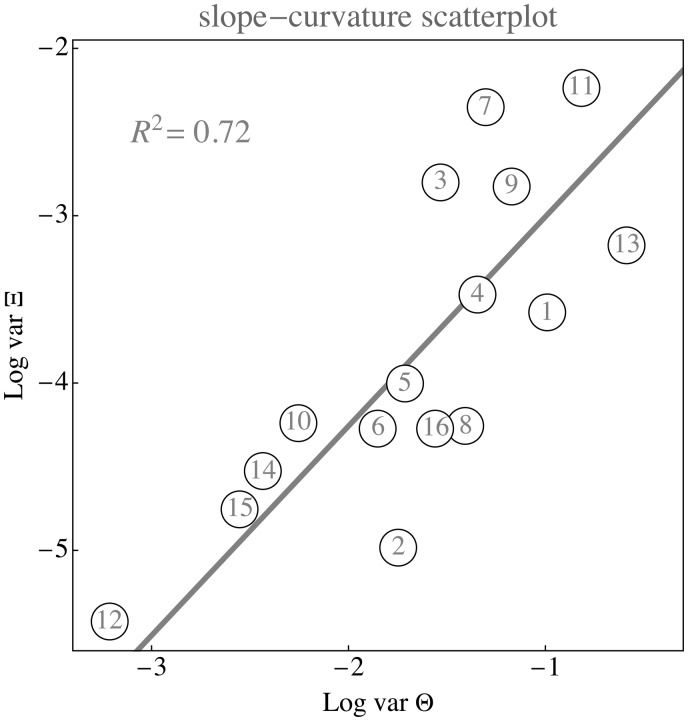
Correlation plot of the natural logarithms of the variances of the parameters Θ and Ξ for the same databases as mined in the previous two figure. The regression line has slope close to unity, indicating a linear dependence. The indices refer to the list of data sources (see Appendix B). In total, this figure is based on $5\times 10^{9}$ RGB samples.

There is a high correlation ($R^{2}=0.72$) between the standard deviation of Ξ and Θ (Figure 16). The best fit is nearly linear (power 1.016…), with a slope is Ψ=0.114…, which apparently is a characteristic universal constant for the sublunar realm.

Although perhaps understood in retrospect (such a dependence is also predicted by Equation (3)), this is evidently a remarkable finding. Most of the variance is in the red–blue, rather than the green–purple. This is due to the autocorrelation length of the articulation spectrum. This general structure is easily reproduced through very simple statistical models that capture the major facts of the ecological optics (see Appendix A).

## Algorithmic Generation of Sublunar Color Gamuts

An obvious method to obtain random instances of a color gamut defined by some database is to simply randomly sample from the database. No generic algorithm needed! However, this involves sampling randomly from hundreds, perhaps thousands of images and randomly sampling pixels from these.

This may well be a viable method if the data source is a single, large image. However, in most cases, an algorithmic synthesis is the only practical way to proceed. It may well be the preferred way too, since it enables the possibility to automatically skip the unavoidable effects of saturation and subthreshold samples.^17^

Since the structure of the sublunar color gamut is well determined and quite simple, it is easy to construct a random generator that will yield as many samples as desired for most purposes. All that is needed is to generate artificial ΛΘΞ triples. Free parameters—within reasonable bounds—are the variances and the nature of the histogram. For a global random gamut generator, one may assume normal distributions of all channels in the physical domain.

It is perhaps most natural to generate the values in the physical domain. Then there are six free parameters, namely, the location and widths of the physical values of the three channels. Thus, the algorithm becomes two tiered. In the first step, one generates random deviates

(11) $$\lambda =N(\mu_{\lambda },\sigma_{\lambda }),\theta =N(\mu_{\vartheta },\sigma_{\vartheta }),\xi =N(\mu_{\xi },\sigma_{\xi })$$

where N(μ,σ) is a random normal deviate of mean *μ* and standard deviation *σ*. At the next step, one calculates

(12) $$\begin{array}{c} (\begin{array}{c} \varrho \\ \chi \\ \beta \end{array})=T^{-1}(\begin{array}{c} \lambda \\ \theta \\ \xi \end{array})\text{where}T^{-1}=\frac{1}{2}(\begin{array}{ccc} 3 & 1 & -2\\ 0 & 2 & 4\\ 3 & -3 & -2 \end{array}) \end{array}$$

and, finally,

(13) r=Ω(ϱ),g=Ω(χ),b=Ω(β)

This may yield apparently *very* different RGB histograms. Starting values for the parameters may be obtained from the analyses of examples.

**Figure 17.**
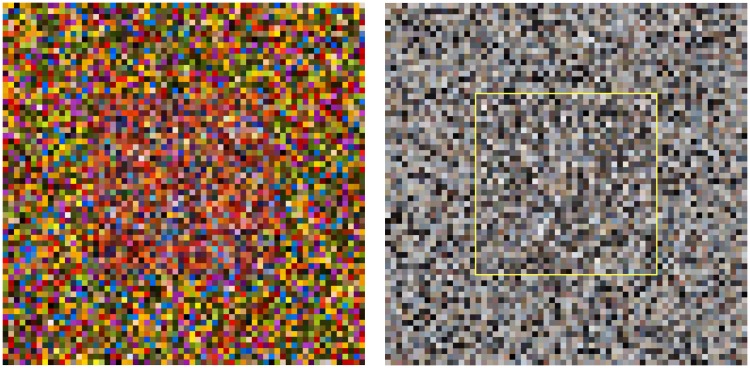
The large square is filled with simulated color samples, whereas the central square inset is filled with actual database samples. The inset has been outlined at right, because in this case (the pebbles image) the simulated gamut cannot be discriminated from the true one. The case of the parrots image (left) is expected to be about “worst case” and indeed, the inset square can be discriminated even without the outline.

In most cases, this will almost perfectly simulate samples from the actual image or database (Figure 17 left for the pebbles image). Exceptions are cases of very inhomogeneous data sources (Figure 17 right for the parrots image). However, even in these cases, the results may well be acceptable for many purposes.

Note that the functions Ω, $\Omega^{-1}$ model mutually extremely diverse types of physics, ranging from something like Kubelka–Munk theory of radiative propagation in layered turbid media to photoelectronic imaging. The parameter that sets the overall level is $\mu_{\lambda }$, whereas the variety of different levels in the scene is captured by $\sigma_{\lambda }$. The parameters $\mu_{\vartheta },\mu_{\xi },\sigma_{\vartheta },\sigma_{\xi }$ model the spectral articulation. Typically $\sigma_{\vartheta }$ dominates the articulation, it is the slope of the SA spectrum. It controls the red–blue spread. The parameter $\sigma_{\xi }$ tends to be of least importance. It sets the curvature of the SA spectrum, controlling the green–purple spread.

Automatic digital cameras are designed to set $\mu_{\lambda }$ to a standard level (e.g., the gray card level) and to set $\mu_{\vartheta }$ and $\mu_{\xi }$ to zero (the automatic “white balance”). There might even be an attempt to control $\sigma_{\lambda }$ (the “contrast”), although this is less common.

Thus, the most informative data is in the three parameters $\left\{\sigma_{\lambda },\sigma_{\vartheta },\sigma_{\xi }\right\}$. This triple is useful as a global spectral signature for the gist of the database.

**Figure 18.**
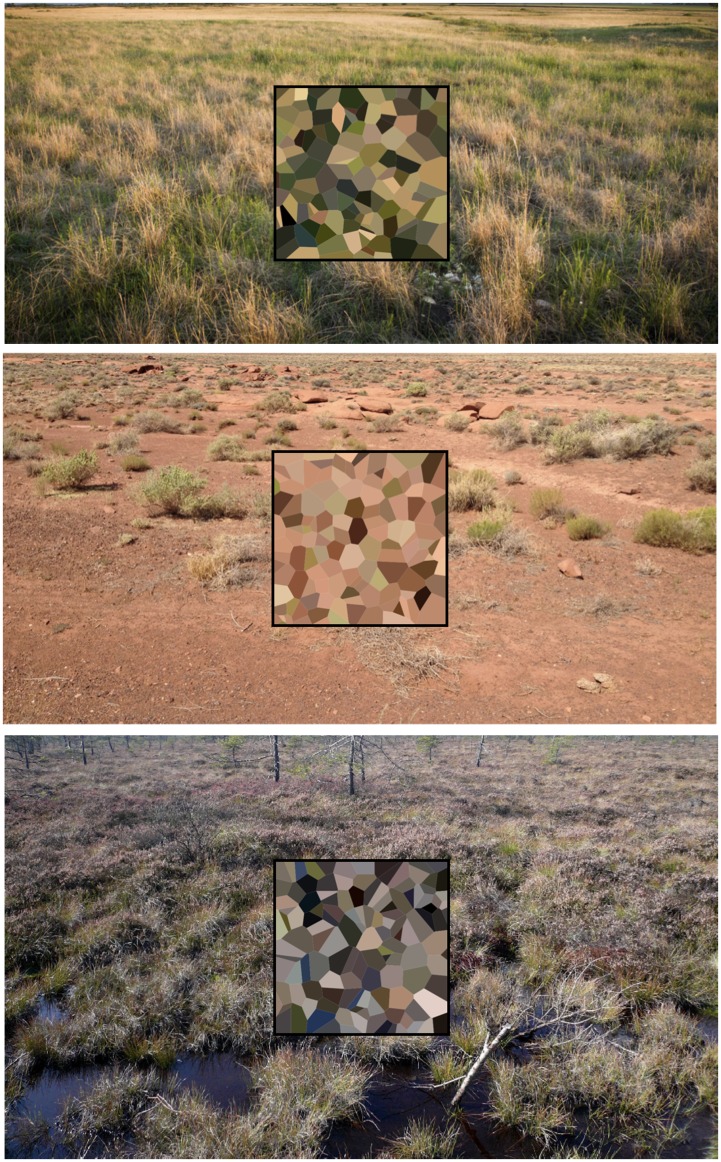
Photographs of the prairie, the Arizona desert and the black moor with insets filled with artificially generated samples based on the statistical analysis of the images.

In applications, one would estimate the parameters from a fiducial set of images, like done in the previous section. It is even a reasonable proposition to estimate parameters from a single, large image. A simple application might be to find a generator for typical terrain colors for use in military camouflage. All that is needed is to provide representative images. In Figure 18, three instances are shown. All conform closely to the assumptions (prairie image Z=2.8, Arizona desert Z=9.6, black moor Z=11.2). Note how the camouflage colors indeed neatly represent the terrain colors.

**Figure 19.**
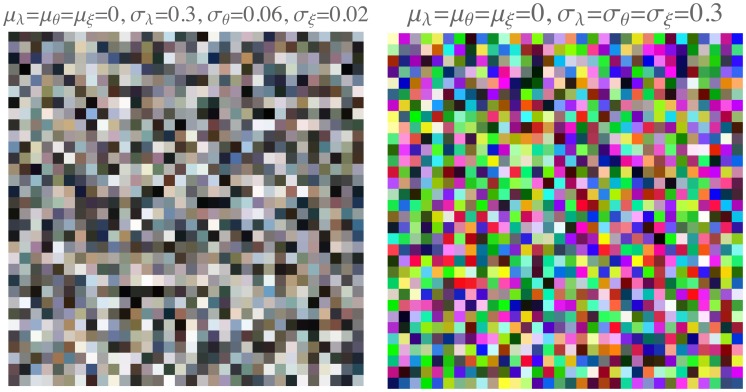
Two random gamuts, obtained with different parameter settings. At left a gamut that might well belong to the sublunar realm and at right a clearly “alien” gamut.

In cases, an “alien” effect is aimed at (like in SF movies), parameters can be assigned more freely, or indeed almost arbitrarily. A simple example is to set $\left\{\mu_{\lambda },\mu_{\vartheta },\mu_{\xi }\right\}$ to zero and $\left\{\sigma_{\lambda },\sigma_{\vartheta },\sigma_{\xi }\right\}$ all to the same value, chosen such that the RGB histograms become approximately flat. Then the RGB covariance matrix will be roughly proportional to the unit matrix, *very much unlike* the typical form for the sublunar. An array of sampled colors looks “garish” and unlike anything you might expect to find in nature. An example is shown in Figure 19 at right. The RGB covariance matrix for this sample is $C_{\mathrm{RGB}}=(\begin{array}{ccc} 98 & 6 & 16\\ 6 & 100 & 3\\ 16 & 3 & 96 \end{array})$. In the same figure (Figure 19) at left is a sample with parameters that might belong to the sublunar. In this case, the RGB covariance matrix is $C_{\mathrm{RGB}}=(\begin{array}{ccc} 98 & 95 & 91\\ 95 & 97 & 96\\ 91 & 96 & 100 \end{array})$. The alien sample differs from the sublunar sample in various ways, but these are perhaps most striking:

—almost all colors are far away from the achromatic axes;
—there is an overdose of saturated greens and purples.

**Figure 20.**
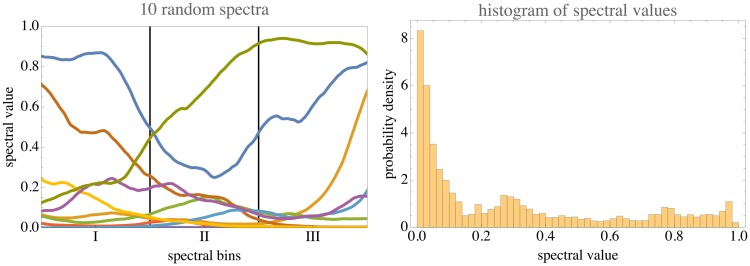
At left, 10 random spectra from the model. The parameter *τ* taken equal to the bin width. The SA power spectrum varies with the inverse square of the frequency. At right, a histogram based on a thousand of such spectra, pooled across wavelengths.

Thus, the algorithm offers a very wide range of readily parameterized color gamuts, which renders it useful for vision research.

The algorithm is sufficiently simple that an interactive developing environment is not hard to implement, allowing a designer to arrive at desirable ΛΘΞ values through an intuitive interface.

## Discussion

We discuss three major topics, the ecological optics of the sublunar realm, the consequences of the generic structure of the SA spectrum for the understanding of the structure of the human visual sense from an evolutionary perspective, and possible applications in computer graphics and image processing.

### Ecological Optics of the Sublunar

Sublunar color gamuts have a simple structure that is invariant over mutually very different domains. This is the case because they all derive from a few generic properties of ecological physics. The main facts of relevance are the narrowness of the visual window and the extent of the SA spectrum autocorrelation length.

Taking account of the mapping of essentially linear physical interaction domains to the observation domain greatly simplifies the descriptions. The six parameters $\left\{\mu_{\lambda },\mu_{\vartheta },\mu_{\xi },\sigma_{\lambda },\sigma_{\vartheta },\sigma_{\xi }\right\}$ typically suffice to characterize the empirical observations of diverse domains. Estimating these parameters from a set of typical images yields useful generic descriptions of these domains. It is likely to be more productive and useful than the conventional methods of acquiring a necessarily rather limited set of reflectance spectra and characterizing these via principal components analysis. The latter is especially problematic in the observation domain because linear combinations of the principal components often assume nonphysical, negative values.

The constant Ψ=0.114… appears to be a universal constant for the sublunar domain. It specifies how fast the autocorrelation of the articulation spectrum falls off with the width of the visual band. Most important deviations from this global pattern—seen from a phenomenological perspective—are the “sky colors” and the colors due to atmospheric perspective. Changes in illumination—be it changes in mere radiative power or (slight) changes in spectral distribution (say from sunlight to skylight)—will hardly imprint themselves on the covariances used in this study.

They will merely make the environment appear a little lighter or darker and will most likely contribute a trend to normality in all channels. Finally, outliers on smallish spatial scales are generally due to flowers, butterflies, some minerals, and on a slightly broader scale human artifacts like paints and so forth. Such outliers are unlikely to be of much consequence, due to their relative scarcity.

Depending on one’s aims, it may be of interest to refine the statistics. Obvious targets are the deviations from normality of the ΛΘΞ distributions. Since the precise form of the sigmoid function is arbitrary, one may force the Λ distribution to normal form. Then the deviations from normality of the Θ and Ξ channels become meaningful parameters. They are likely to be domain specific.

Our results are in accordance with Attewell and Baddeley (2007) who measured full spectral reflectance functions in the field. However, these authors remain in the reflectance domain and do not consider spectral correlations.

Full (high-resolution) spectral imaging (Ruderman, Cronin, & Chiao, 1998) also yields results close to these found here. Their estimation of the precise opponent directions is similar to ours. Apparently true hyperspectral imaging (a major chore) does not yield much beyond mere RGB crowd sourcing. This is only to be expected.

### Articulations of the SA Spectrum and the Human Visual Sense

As we have shown, the opponent directions as phenomenologically identified by Hering (1920), turn out to derive from ecological physics. Their dominant appearance in the ecological optics is due to the nature of the spectral articulations. The structure of the SA spectrum in a three-bin representation is characterized by the SA spectral slope and the SA spectral curvature, two properties that are expected to be mutually uncorrelated, whereas the first order (slope) is expected to dominate the second order (curvature). This gives rise to the dominant eigendirections found in essentially any image of the sublunar realm.

Thus, Hering’s opponent colors, identified from a phenomenological analysis, may well have resulted from an evolutional drive toward the informationally desirable decorrelation of sensor channels.

In view of the empirical value of Ψ, it appears a good design objective to limit the biological spectral resolution to a mere two or three degrees of freedom, as indeed resulted from evolutionary pressure. Because the correlation length of the SA spectrum is of the order of a spectral bin width, there is hardly a pressure for tetrachromacy from an ecological perspective.

Thus, both trichromacy and opponency appear as adaptations to the ecological optics of the sublunar realm.

That opponent channels serve to effectively decorrelate the spectrally related optics nerve activity was already suggested by Buchsbaum and Gottschalk (1983). However, these authors effectively find the principal components of the color matching functions, not the spectral covariance. Thus, they implicitly treat the spectrum as white noise and the correlation structure as due to the mutual overlap of the color matching functions. This is categorically different from our perspective. However, from a biological perspective, the color matching functions are evolution’s answer to the spectral correlation, so the similarity of results is perhaps not a miracle, though certainly far from trivial.

Technology arrives at similar insights by a process of successive improvements driven by practical constraints. That the RGB channels tend to be highly correlated was already used in the 1953 (second) NTSC standard for analog TV. The luminance–chrominance encoding was already invented in 1938 by Georges Valensi.^18^ The FCC version of the NTSC standard uses an intensity signal Y=0.30R+0.59G+0.11B (which may serve to drive monochrome receivers) and chrominance signals I=0.599R-0.2773G-0.3217B and Q=0.213R-0.527G+0.3121B, thus the *I* signal is a red–cyan and the *Q* signal is a magenta–green opponent signal. The *Y* signal is allotted a bandwidth of 4Mhz, the *I*-signal 1.3 Mhz, and the *Q* signal 0.4 Mhz, this evidently reflects the typical covariances found in RGB images. The YIQ encoding is often construed as fitting the human visual system, in reality it fits the covariance of the spectra of the sublunar realm.

### Applications in Computer Graphics and Image Processing

Random gamut generators are likely to find applications in computer graphics, where it is often desirable (for instance in synthesizing various landscapes) to generate large numbers of instances of colors belonging to a restricted setting in an intuitively parameterizable way. Of course, such reflectance factors can be combined with various spectral illuminants to transform the gamut, say from a noon to a later afternoon setting.

Such color generators may also find application in interior design, textiles design, and so forth. They yield color gamuts that can be made to perfectly fit any well-defined environment in a simple, principled manner.

Although this exercise in capturing the “color gamuts of the sublunar” is possibly useful, there remain—of course—numerous loose ends. Some are due to the extreme generalizations that had to be made. As a consequence, numerous important effects of ecological optics were fully ignored. Perhaps most blatantly, no account was taken of the effects of geometry, obviously of major importance to the irradiation of the scattering surfaces and thus to the radiance scattered to the camera or eye. Such issues become relevant in applications of machine vision and image processing. Examples include image segmentation (Comaniciu & Meer, 1997) and recognition on the basis of color gamuts (Gevers & Smeulders, 1999). Here, more intricate statistical analysis, as mentioned above, may well turn out to be useful.

## Conclusions

So what are the gamuts of the sublunar like? In view of the correlations shown in Figure 16 and perhaps surprisingly, a rather specific answer is possible. First of all, they are quite gray, touches of hues being special—thus biologically important. The variations are dominated by monochrome contrast. The major chromatic variations are in the range from orange to greenish–blue, or—as painters have it—“warm” to “cool.” Minor variations are in the range green to dark purple, what painters sometimes denote as “moist” to “dry” (Benson, 2000).

**Figure 21.**
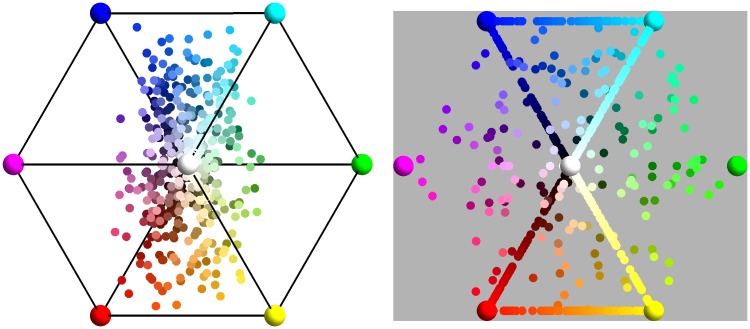
Left: A thousand random RGB samples from the model. The parameter *τ* taken equal to the bin width. Right: A thousand random RGB samples from the model in the case of zero shift and large scaling. The spectra are approximately degenerated to random telegraph waves. Note that the random RGB colors accumulate on six of the edges of the cube, the other edges remaining unpopulated. These colors are Goethe’s edge colors (G. *Kantenfarben*).

The big picture is evidently *dominantly GRAY contrast with some red–blue and even fewer green–purple variations.* This is largely due to basic physics (especially clear in Figure 21 of Appendix B) and constraints of human physiology which—by way of evolution—most likely have been shaped by the ecological structure itself.

## Supplementary Material

Supplementary material

Supplementary material

Supplementary material

Supplementary material

Supplementary material

Supplementary material

Supplementary material

Supplementary material

Supplementary material

Supplementary material

## Supplemental Material

The online appendix movies are available at http://journals.sagepub.com/doi/suppl/10.1177/2041669517733484.

## Notes

1 The R, G, and B values are taken to be confined to the range [0,1].

2 Note that the colorimetric literature would use “magenta” instead of “purple” and “cyan” instead of “turquoise.” We prefer the more common terms used in the visual arts and so forth.

3 The notion of “Umwelt” derived from biology, or, more precisely ethology. One means that part of the sublunar realm that is causally involved in stimulating an animal’s senses and that part of an animal’s effectors that may change the environment. Different animals tend to have different Umwelts, even though they coexist in a single physical environment.

4 Two spectra that cannot be visually discriminated differ by an element of the null-space. There are infinitely many of such “metameric blacks.”

5 Linear projections conserve convexity and central symmetry.

6 The normalized covariance matrix is $\begin{array}{c} (\begin{array}{ccc} 100 & -22 & -24\\ -22 & 86 & -26\\ -24 & -26 & 89 \end{array}) \end{array}$.

7 The CIE xy-chromaticity coordinates are {0.6084,0.3916} for the red, {0.2219,0.6632} for the green, and {0.1442,0.0574} for the blue, whereas the Y-coordinates are in the ratio 38:55:7. The chromaticity of the white point is {0.3138,0.3310}.

8 Of course, one could easily make it a “true octave” by taking the tails of the color matching functions into account. We would say that “somewhat less than an octave” is the most useful statement.

9 This is a generic example, of course. In practice, the diagonal elements often differ somewhat and so forth. The main conclusions from the example remain unchanged when the matrix is somewhat perturbed. In contradistinction, the “default” case mentioned above gives rise to a roughly diagonal matrix.

10 The reflectance factor defined as the ratio between a radiometric reading for sample surface and the reading for a white card held in the same spatial attitude and location.

11 Purely phenomenologically one might expect red–cyan, green–magenta and blue–yellow as opponent colors, for these are the only complementary cardinal color pairs and there is no a priori reason to choose between them. Picking any two from these three requires a reason, which appears to be lacking.

12 Kubelka–Munk theory is not essentially involved with spectral descriptions. Kubelka-Munk theory is important for other reasons that are explained below.

13 Imaging is not essentially involved with spectral descriptions. Imaging is important for other reasons that are explained below.

14 In Google Image “Search Tools” one has “Any size,” “Large” and “Medium.” Medium size is around 512 × 512–pixels.

15 In this section, all channel values are given as rounded values between 0 and 100. This is preferable from typographic perspectives and reads easily. From a coding perspective, the range 00–99 (all two-digit numbers, in contradistinction to 0–100, which has one, two- and three-digit numbers) is perhaps most convenient.

16 Often done automatically by the in-camera firmware, otherwise usually by some image correction process, often automatic, sometimes manually (the “levels” or “curves” methods of programs like Adobe’s Photoshop). One expects r:g:b≈1:1:1 on the average.

17 In most images, one finds an accumulation of debris at the limits. This is due to radiance levels exceeding the dynamic range of the sensor. In the case of the (typical) byte (0–255) representation it is always a wise decision to limit the range to 1–254, perhaps even better to 5–250. This has the additional advantage that the inverse sigmoid transformation will never meet an argument it cannot handle.

18 FR patent 841335, Valensi, Georges, “Procédé de télévision en couleurs”, published 1939–05–17, issued 1939–02–06.

## Appendix A

### Simulation of Sublunar Spectra

Using the basic physical insights that the spectral envelope is statistically translation invariant along the spectral axis and that its autocorrelation function is peaked, falling symmetrically and monotonically off toward both sides, one readily simulates the basic structure of the sublunar colors.

Since we have no data on the shape of the autocorrelation function, we simply use a Laplace distribution (note that $\int_{-\infty }^{+\infty }L(x,\tau )\text{d}x=1$)

(14) $$L(x,\tau )=\frac{{\text{e}}^{-\frac{\left|x\right|}{\tau }}}{2\tau }$$

with Fourier transform

(15) $$S(f)=\frac{1}{\sqrt{2\pi }}\frac{1}{1+f^{2}\tau^{2}}$$

Other peaked functions, like the Gaussian, yield very similar results. It seems possible that more homogeneous data might render it reasonable to attempt to differentiate further, we do not attempt that here.

Random spectra are computed by convolving this kernel with white noise, generated as a list of normal deviates. This yields signals with a SA power spectrum that falls off with the square of the SA frequency. (Typically the convolution is computed via FFT, which assumes periodic boundary conditions. Thus, it is important to compute spectra over a much longer interval than will be used in an observation. Failing to do so introduces a spurious correlation between the outermost bins.)

The resulting spectra are generated in the physical domain. Before transforming to the observation domain, one may arbitrarily scale and shift the functions so as to obtain a fairly typical histogram of the values. The exact choice is of hardly any relevance to the eventual result. However, it increases the illusion of “reality” of the simulation.

The resulting spectral samples could hardly be distinguished from a set of a few thousand observations from the sublunar realm (Figure 20). This is perhaps due to an overdose of detail. Translation invariance and the overall nature of the autocorrelation function, or, equivalently, the mean spectral articulation power spectrum, are the determining factors.

The covariance matrices of this type of simulation can be readily appreciated from about a thousand samples or so. A million samples are easily computed in reasonable time, but are really overkill. In this case

(16) $$\begin{array}{c} C_{\Lambda \Theta \Xi }=(\begin{array}{ccc} 100 & 1 & 7\\ 1 & 28 & 1\\ 7 & 1 & 7 \end{array}) \end{array}$$

thus the ratio of the opponent variances is 4. The result depends mainly on the choice of the correlation length τ (here taken equal to the bin width), relative to the bin width.

It is of some interest to consider the distribution of RGB values in the RGB cube (Figure 21 left). As expected, there is a much wider range in the orange–greenish blue than in the green–purple. The distribution over the white–black axis is mainly due to the setting of offset and width in the physical domain, they hardly affect the resulting opponent covariance matrix.

Setting the shift to zero and the scaling very high, the generated spectra become approximately random telegraph waves. This is the limit we have considered in an earlier, mainly theoretical, investigation (Koenderink, 2010b). The RGB colors largely concentrate on the edge color series black–red–yellow–white and white–turqoise–blue–black. The green–purple dimension is hardly populated (see Figure 21 right). The covariance matrix becomes

(17) $$C_{\Lambda \Theta \Xi }=(\begin{array}{ccc} 100 & 0 & 8\\ 0 & 33 & 0\\ 8 & 0 & 11 \end{array})$$

thus the ratio of the opponent variances is 3. The material discussed here is available on the website of the publisher as Mathematica Notebooks, that can be read with the free Mathematica Reader, or run in the Mathematica application. (For Mathematica see https://www.wolfram.com/mathematica/).

## Appendix B

### The Data Sources

The data sources range from single images to databases containing over 5,000 images:

Overall, about 10,000 images were used in a variety of sizes. The square sizes (the “square size” is defined as the rounded square root of the total number of pixels in the image; it is a convenient measure, since aspect ratios vary all over the place) range from 256 to almost 3,000 (quartiles {256, 410, 1483}). This yielded a total data volume of $4.84\times 10^{9}$ pixels. The distributions over the items are obviously very uneven, the range is $1.96\times 10^{6}$–$3.88\times 10^{9}$, the quartiles $\left\{6.49\times 10^{6},2.09\times 10^{7},2.78\times 10^{7}\right\}$.

The images are rather uniform data sources, they were selected for that. The databases are also uniform in a sense, although there is quite a bit of variation. Overall, it makes sense to treat the images and the databases (which may contain numerous images) as individuals in the analysis.

**Table:**

Index	Name	#Items	Size	Data volume
1	MIT db: Coast	361	256	23 658 496
2	Image: Desert soil	1	2827	7 990 272
3	MIT db: Forest	329	256	21 561 344
4	Image: Heath	1	1399	1 958 400
5	MIT db: Highway	261	256	17 104 896
6	MIT db: InsideCity	309	256	20 250 624
7	Leeds db: Butterflies	762	662	333 473 896
8	MIT db: Mountain	375	256	24 576 000
9	MIT db: OpenCountry	411	256	26 935 296
10	Oxford db: Oxford buildings	5064	875	3 878 103 040
11	Oxford db: Flowers	1360	565	434 358 354
12	Image: Pebbles	1	2234	4 990 464
13	Image: Prairie	1	1648	2 714 340
14	Image: SchwarzesMoor	1	1567	2 455 864
15	MIT db: Street	293	256	19 202 048
16	MIT db: Tall building	357	256	23 396 352
