# Supplementary material for: Colors of the Sublunar
Source: Iperception. 2017 Sep 29;8(5):2041669517733484. doi: 10.1177/2041669517733484 (PMC5624368; doi:10.1177/2041669517733484)

Appendix A: Simulated spectra

The spectal radiant power noise spectrum

Next we defined a spectral kernel based on the Laplace distribution of parameter tau:

(Note: The choice of the Laplace distribution is arbitrary.)

```
laplaceDistribution[x_, tau_] := Exp[-Abs[x] / tau] / (2 tau);
TraditionalForm[laplaceDistribution[x, τ]]
```

$$\frac{e^{-\frac{|x|}{\tau}}}{2\tau}$$

```
Plot[
 laplaceDistribution[x, 1],
 {x, -5, 5},
 PlotStyle → {Red, Thick},
 Filling → Axis,
 PlotLabel → "Laplace PDF"
]
```

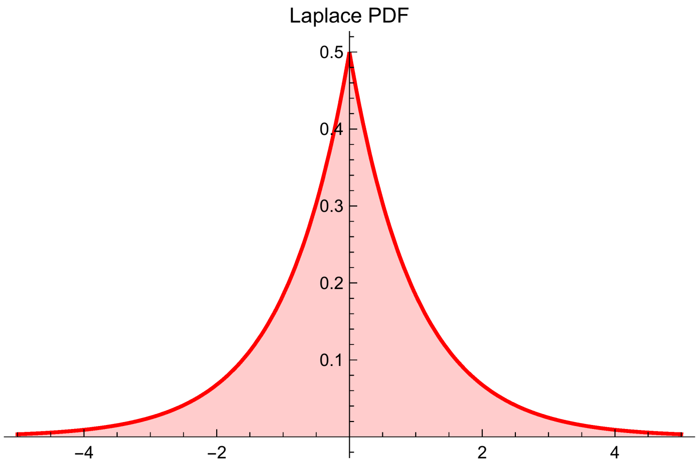

The Laplace distribution is normalized:

```
Integrate[laplaceDistribution[x, tau],
 {x, -Infinity, Infinity}, Assumptions → tau > 0]
```

1

Its fourier transform is close to a one over frequency squared spectrum:

```
FourierTransform[laplaceDistribution[x, τ], x, f, Assumptions → τ > 0] //
 TraditionalForm
```

$$\frac{1}{\sqrt{2\pi}\,(f^2\tau^2+1)}$$

```
LogLogPlot[(1/(Sqrt[2 π] (1 + f^2 tau^2)) /. tau → 1), {f, 1, 1000}, Filling → 10^-7]
```

$$\text{LogLogPlot}\left[\left(\frac{1}{\sqrt{2\pi}\,(1+f^2\,\text{tau}^2)}\ /.\ \text{tau}\to 1\right),\ \{f,1,1000\},\ \text{Filling}\to 10\wedge-7\right]$$

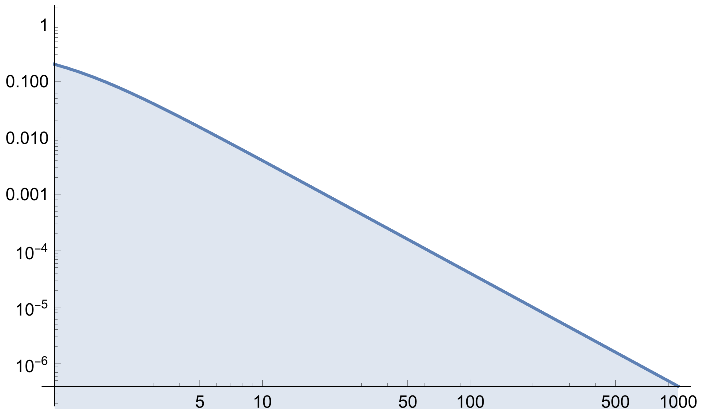

Constants of range

We consider a spectral range of width 1, sampled in m bins:

```
numBins = 256;
```

Computations are done over a much longer width:

```
extendedRange = 9.0;
```

this will be necessary because we use FFT methods that treat the width as having periodic boundary conditions.
So we use a long interval and use only part of that.
In the end we throw away the ranges (0,3) and (6,9), whereas we will use the ranges (3,4), (4,5) and (5,6) for the red, green and blue spectral parts.
This effectively removes the influence of the periodic nature of the FFT.

The total number of samples n and the binwidth dx thus become:

```
n = Round[extendedRange * numBins];
binWidth = extendedRange / (n - 1);
```

Random spectra

We need the fourier spectrum of the Laplace density. This involves minor juggling. You will probably do that in MatLab or Python. It is standard (you can skip the next code entry, it is conceptually irrelevant):

```
τ = 1.0;
kernel = Table[If[λ ≤ extendedRange / 2, Exp[-λ / τ],
    Exp[- (extendedRange - λ) / τ]], {λ, 0, extendedRange, binWidth}];
kernel = kernel / Total[kernel];
kernelSpectrum = Fourier[kernel];
```

Here is some Gaussian white noise:

```
noise = Table[Random[NormalDistribution[0, 1]], {λ, 0, extendedRange, binWidth}];
ListPlot[noise]
```

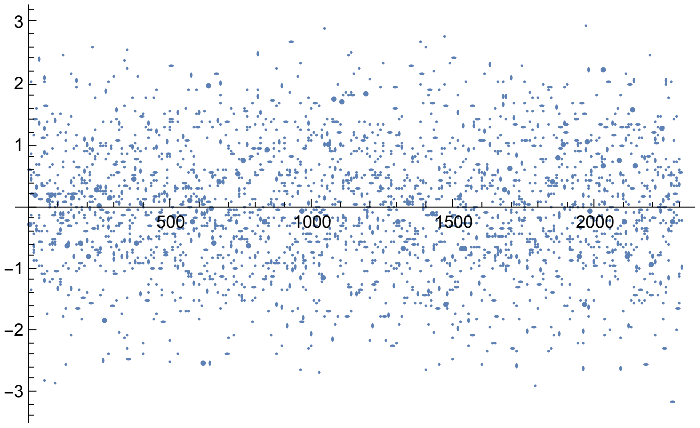

And here the noise is filtered by the kernel:

```
filtered = Chop[InverseFourier[Fourier[noise] kernelSpectrum] Sqrt[n]];
ListPlot[filtered]
```

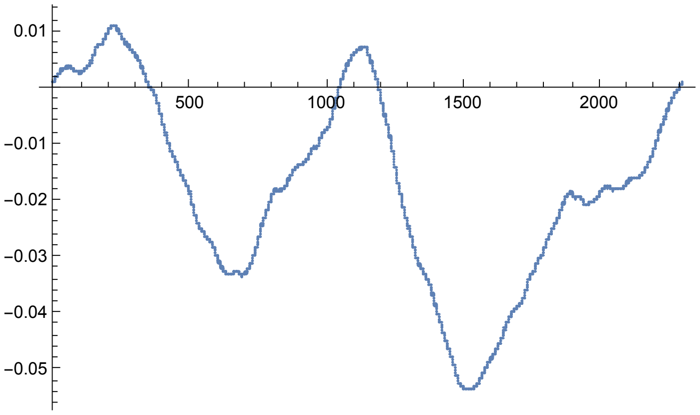

The ogive function mapping to the [0,1]-range

We use a simple and fast method:

```
ogive[x_] := Exp[x] / (Exp[x] + Exp[-x])
Plot[ogive[x], {x, -5, 5}, PlotStyle → {Black, Blue}, Filling → Axis]
```

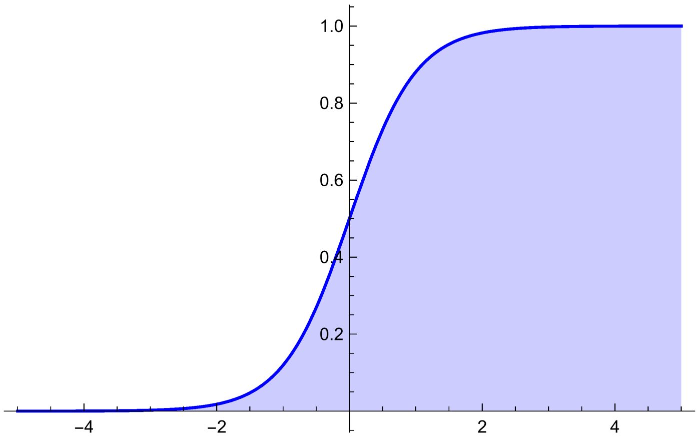

Remember: essentially all such functions are born equal.

You can substitute your favorite here, it will make no difference.

Sample spectra

In order to use the ogive transfer we need to set some location and range:

```
amplitude = 40.0;
offset = -1.0;
```

Here is a function that yields an instance of a randiant power spectrum:

```
sample[] :=
  ogive[
   offset + amplitude *
     Chop[
      InverseFourier[
        Fourier[Table[Random[NormalDistribution[0, 1]],
           {λ, 0, extendedRange, binWidth}]] kernelSpectrum
       ] Sqrt[n]
     ]
  ];
```

Here are ten samples:

```
ListPlot[Table[sample[], {10}], PlotRange → All, Joined → True, ImageSize → 500]
```

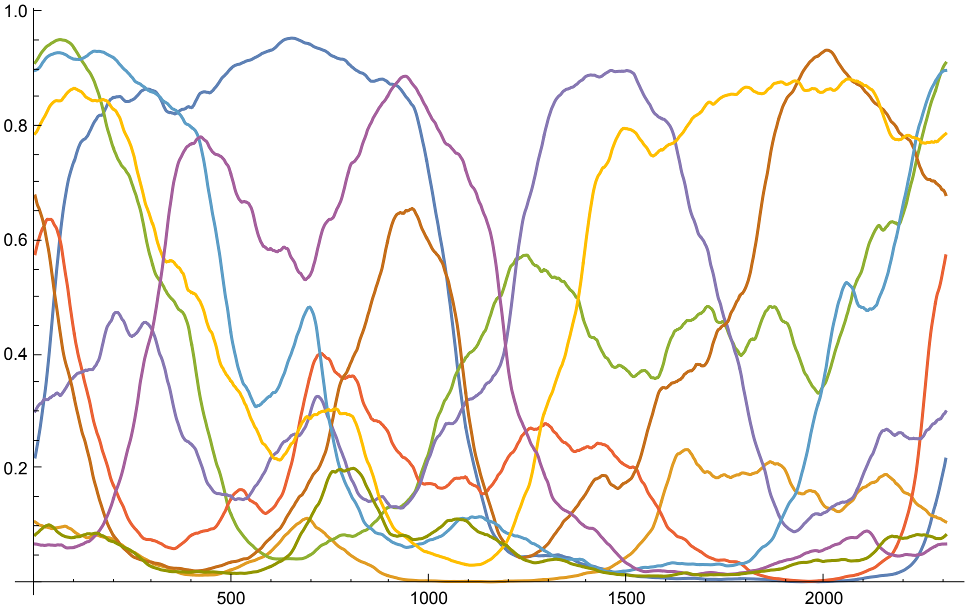

Here is a histogram:

```
Histogram[
 Flatten[Table[sample[], {10}]],
 "FreedmanDiaconis",
 "PDF",
 Axes → False,
 Frame → True,
 FrameStyle → Table[{Thin, Black}, {4}],
 FrameTicksStyle → Table[{Thin, Black}, {4}],
 BaseStyle → Directive[FontFamily → "Times", FontSize → 20],
 FrameLabel → {{"probability density", None}, {"spectral value", None}},
 PlotLabel → "histogram of spectral values",
 ImageSize → 500
]
```

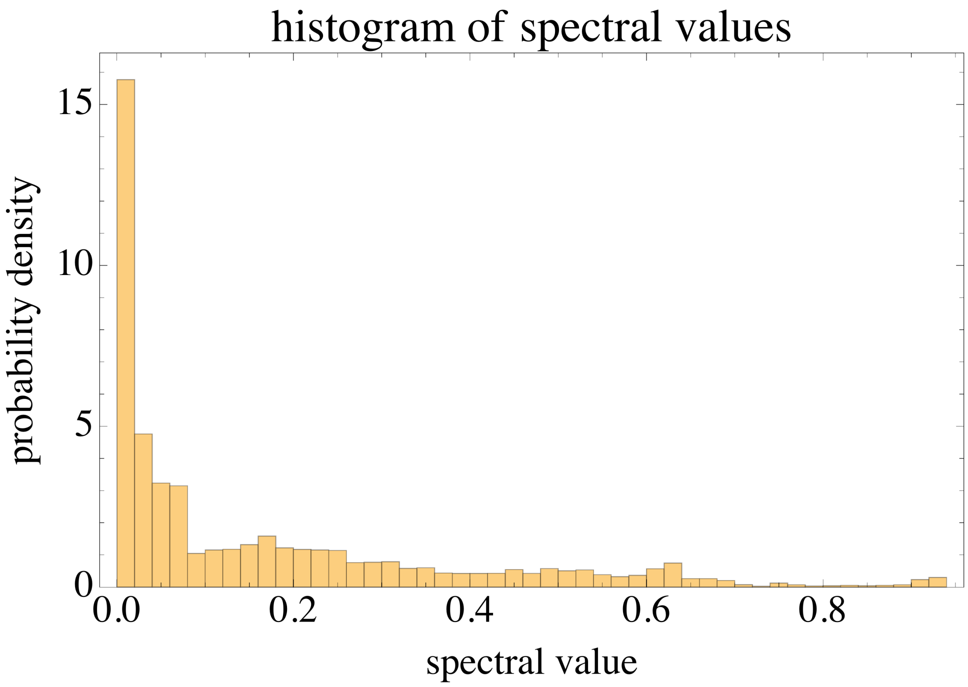

Sampling RGB spectral ranges

We discard ranges of width 3 from either end of the total spectral range of 9, this gets rid of the periodicity imposed by the FFT. We are left with a center range of width 3, we split this into three ranges of length 1 each: These are taken as the R, G and B “parts of daylight” ranges.

Here is a function that achieves this:

```
ListPlot[
 Table[
  Transpose[{Table[λ, {λ, 0, extendedRange, binWidth}], sample[]}],
  {10}
 ],
 PlotRange → {{0, 3}, {0, 1}},
 Joined → True,
 PlotStyle → AbsoluteThickness[4],
 Axes → False,
 FrameStyle → Table[{Thin, Black}, {4}],
 FrameTicksStyle → Table[{Thin, Black}, {4}],
 Frame → True,
 FrameTicks →
  {{Automatic, None}, {{{0.5, "I"}, {1.5, "II"}, {2.5, "III"}}, None}},
 Prolog → {Black, Thick, Table[Line[{{i, 0}, {i, 1}}], {i, 1, 2}]},
 BaseStyle → Directive[FontFamily → "Times", FontSize → 20],
 FrameLabel → {{"spectral value", None}, {"spectral bins", None}},
 PlotLabel → "10 random spectra",
 ImageSize → 500
]
```

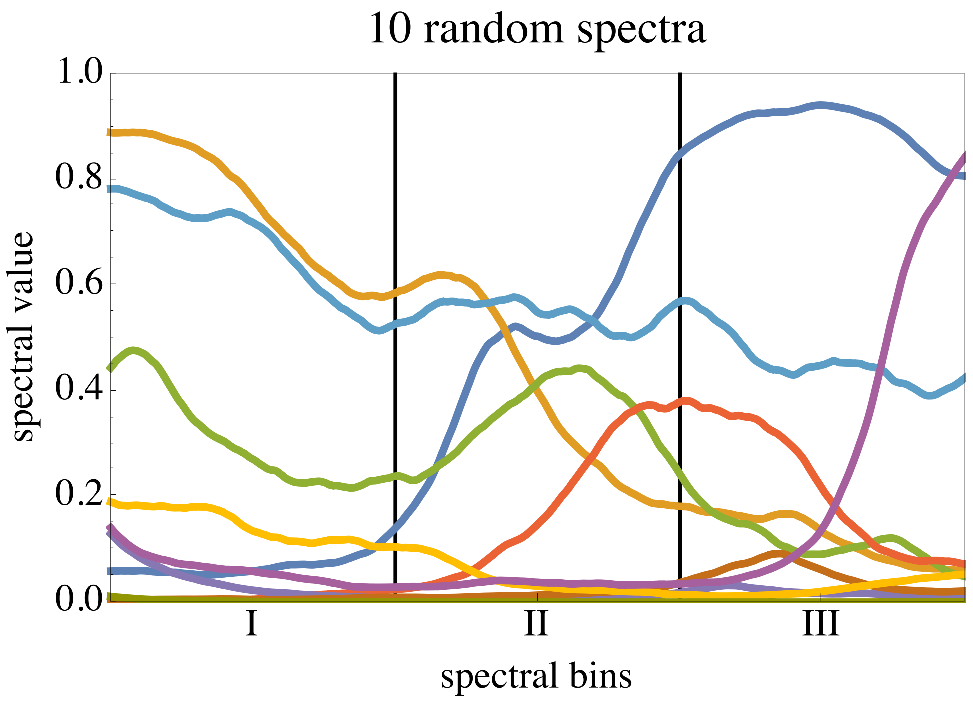

Here we harvest the R, G and B ranges:

```
rgbChannels[aSample_] :=
 Map[
  Mean,
  {
   aSample[[Range[1, numBins]]],
   aSample[[Range[numBins + 1, 2 numBins]]],
   aSample[[Range[2 numBins + 1, 3 numBins]]]
  }
 ]
```

What happens here is that we simply integrate over the ranges I, II and III separately, whereas the other parts of the extended range are simply discarded.

This may seem to be a waste, but it is actually ESSENTIAL.
This is because the fourier transformation treats its interval as periodic.
By discarding both ends we get rid of this spurious periodicity.

Try for yourself, you will notice the importance. If the spectral range was actually periodic (which it is NOT), you obtain very different (WRONG!) statistics!

Sample R, G and B spectra

Here we consider a thousand independent samples.

```
amplitude = 40.0;
offset = 0.0;

numSamples = 1000;
rgbSamples = Table[rgbChannels[sample[]], {numSamples}];

covarianceRGB = Covariance[rgbSamples];
Round[100 covarianceRGB / Max[Flatten[covarianceRGB]]] // MatrixForm
```

$$\begin{pmatrix} 100 & 72 & 38 \\ 72 & 97 & 73 \\ 38 & 73 & 98 \end{pmatrix}$$

Notice the strong correlations between regions I, II and III. However, regions I and III are evidently LESS correlated than either ranges I and II or ranges II and III. This is because we discarderd the outer parts of the extended range.

```
Grid[{Map[Histogram, Transpose[rgbSamples]]}]
```

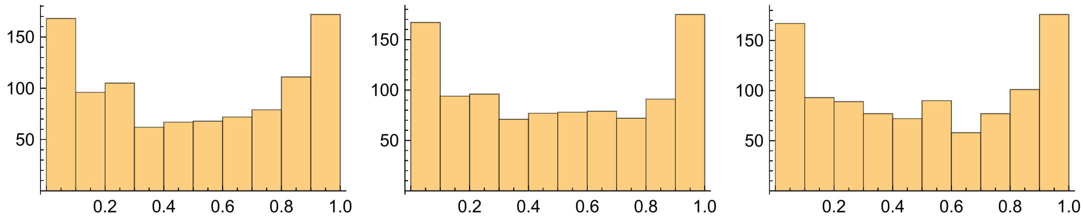

Samples in an opponent basis

```
opponentBasis = N[Map[Normalize, {{1, 1, 1}, {1, 0, -1}, {-1, 2, -1}}]];
opponentChannels[rgbSample_] := opponentBasis.rgbSample
```

```
opponentSamples = Map[opponentChannels, rgbSamples];
covarianceOpp = Covariance[opponentSamples];
Round[100 covarianceOpp / Max[Flatten[covarianceOpp]]] // MatrixForm
```

$$\begin{pmatrix} 100 & 0 & 7 \\ 0 & 28 & 0 \\ 7 & 0 & 6 \end{pmatrix}$$

As expected, the covariance matrix becomes nearly diagonal.

Distribution in the RGB cube

We plot samples in the RGB-cube.
Colors are concentrated about a greenishBlue-orange plane, there are no true greens and purples.
(Green is on the right, purple on the left, view is from the KW-axis.)

We use offset 0 for a symmetrical sample.

```
amplitude = 40.0;
offset = 0.0;
```

```
numSamples = 500;
rgbSamples = Table[rgbChannels[sample[]], {numSamples}];
```

```
Graphics3D[
 {
  FaceForm[],
  EdgeForm[{Black, Thick}],
  Cuboid[{0, 0, 0}, {1, 1, 1}],
  EdgeForm[],
  AbsolutePointSize[8],
  Map[{Apply[RGBColor, #], Point[#]} &, rgbSamples],
  FaceForm[Red], Sphere[{1, 0, 0}, 0.05],
  FaceForm[Yellow], Sphere[{1, 1, 0}, 0.05],
  FaceForm[Green], Sphere[{0, 1, 0}, 0.05],
  FaceForm[Cyan], Sphere[{0, 1, 1}, 0.05],
  FaceForm[Blue], Sphere[{0, 0, 1}, 0.05],
  FaceForm[Magenta], Sphere[{1, 0, 1}, 0.05],
  FaceForm[White], Sphere[{1, 1, 1}, 0.05]
 },
 Boxed → False,
 BoxStyle → {Black, Thick},
 Lighting → "Neutral",
 ViewVertical → {0, 0.5, 1},
 ViewPoint → 1000 {1, 1, 1}
]
```

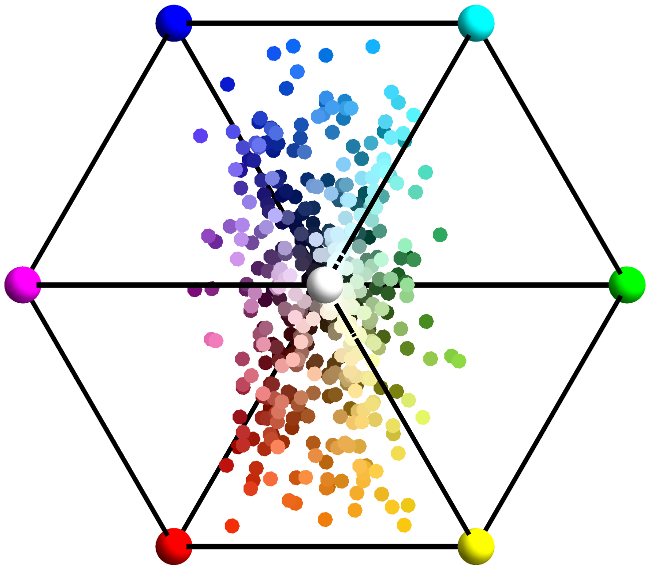

Notice the relative scarcity of greens and purples.

One sees a non-uniform distribution of RGB-colors, despite the fact that the statistics are translationally invariant along the spectrum.

This fact was first noticed by Goethe.

Random telegraph waves

By setting the amplitude very high, the spectra degenerate into approximately random telegraph

waves. This is of some interest, because the Schrödinger optimal colors are random telegraph waves too, albeit of a simple subkind.

```
amplitude = 1 000 000.0;
offset = 0.0;
```

```
sample[] :=
  ogive[
   offset + amplitude *
     Chop[
      InverseFourier[
        Fourier[Table[Random[NormalDistribution[0, 1]],
           {λx, 0, extendedRange, binWidth}]] kernelSpectrum
       ] Sqrt[n]
     ]
  ];
```

These are very degenerate spectra:

```
Grid[
 Table[
  ListPlot[sample[], PlotRange → All,
   Joined → True, Filling → Axis, ImageSize → 200],
  {2}, {3}
 ]
]
```

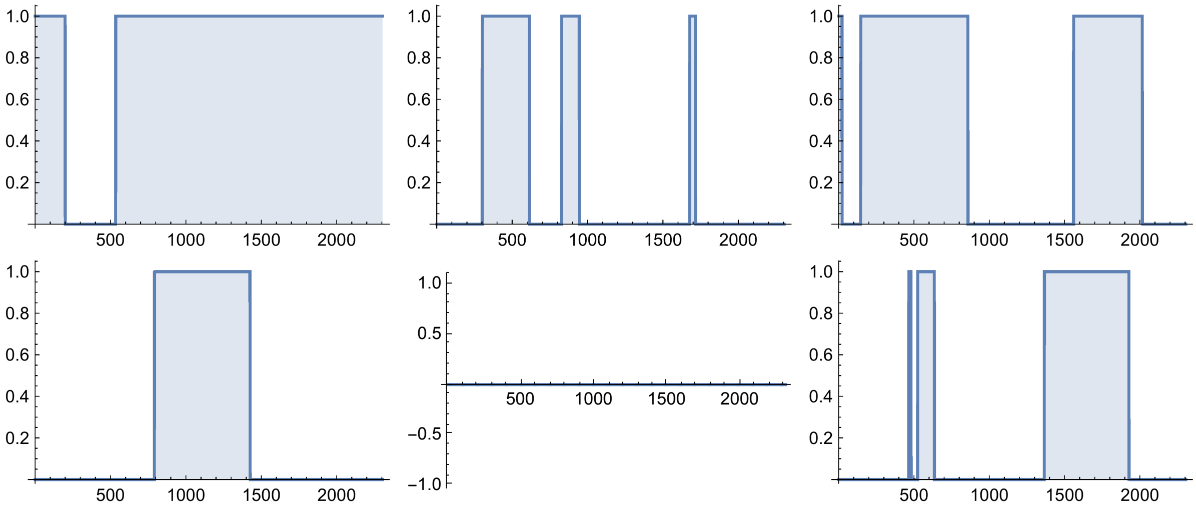

Of course, the histogram becomes very degenerate too:

```
Histogram[
 Flatten[Table[sample[], {10}]],
 "FreedmanDiaconis",
 "PDF",
 Axes → False,
 Frame → True,
 FrameStyle → Table[{Thin, Black}, {4}],
 FrameTicksStyle → Table[{Thin, Black}, {4}],
 BaseStyle → Directive[FontFamily → "Times", FontSize → 20],
 FrameLabel → {{"probability density", None}, {"spectral value", None}},
 PlotLabel → "histogram of spectral values",
 ImageSize → 300
]
```

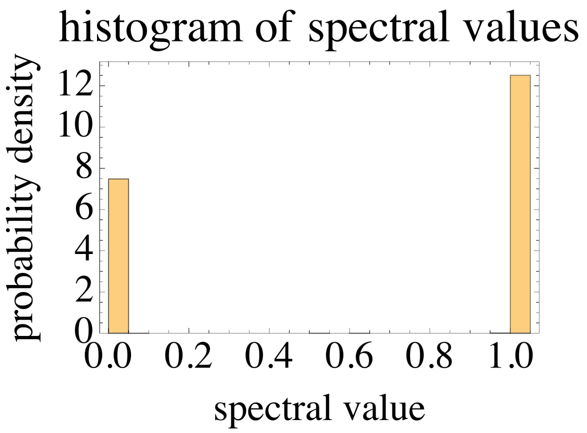

The random telegraph waves straddle the RGB boundaries:

```
Grid[
 Table[
  ListPlot[
   Transpose[
    {Range[1, numBins * 3], sample[][[Range[numBins * 3 + 1, numBins * 6]]]}],
   PlotRange → {All, {0, 1.2}},
   Joined → True,
   PlotStyle → {Black, Thick},
   Filling → Axis,
   FillingStyle → {Black, Opacity[0.5]},
   Prolog → {
     RGBColor[0.5, 0.5, 1], Rectangle[{0, 0}, {numBins, 1.2}],
     RGBColor[0.5, 1, 0.5], Rectangle[{numBins, 0}, {2 * numBins, 1.2}],
     RGBColor[1, 0.5, 0.5], Rectangle[{2 * numBins, 0}, {3 * numBins, 1.2}]
    },
   ImageSize → 200
  ],
  {2}, {3}
 ]
]
```

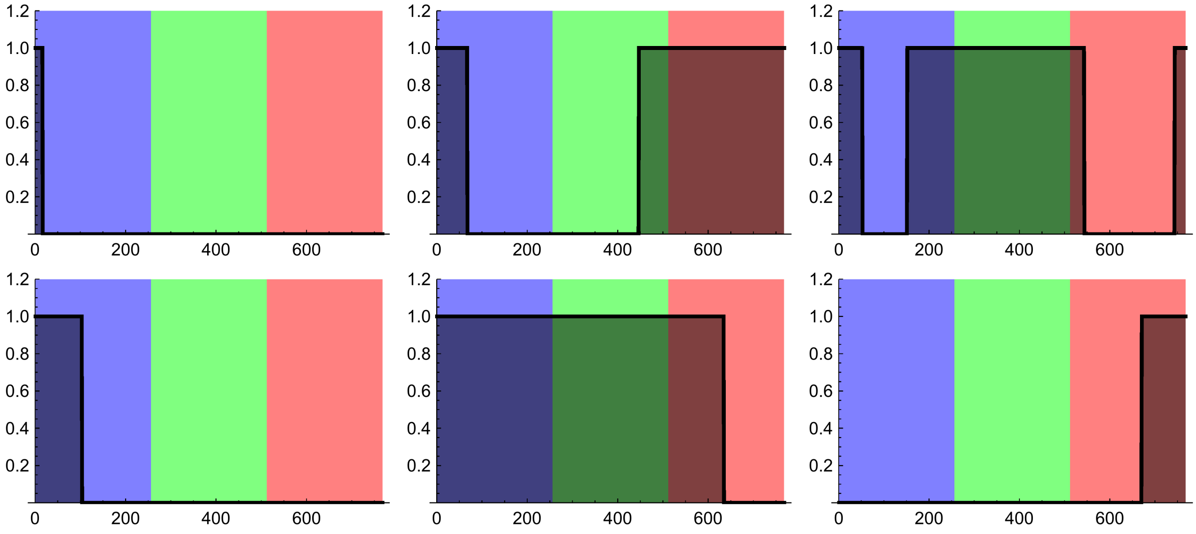

Thus one may get all kinds of RGB-colors. However, some RGB-colors are more frequent than others.

Here is a simulation:

```
amplitude = 1 000 000.0;
offset = 0.0;
```

```
numSamples = 1000;
rgbSamples = Table[rgbChannels[sample[]], {numSamples}];
```

```
covarianceRGB = Covariance[rgbSamples];
Round[100 covarianceRGB / Max[Flatten[covarianceRGB]]] // MatrixForm
```

$$\begin{pmatrix} 99 & 65 & 31 \\ 65 & 99 & 65 \\ 31 & 65 & 100 \end{pmatrix}$$

```
Grid[{Map[Histogram, Transpose[rgbSamples]]}]
```

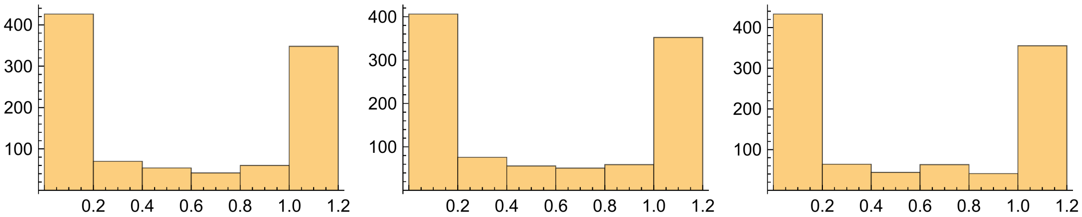


```
opponentSamples = Map[opponentChannels, rgbSamples];
covarianceOpp = Covariance[opponentSamples];
Round[100 covarianceOpp / Max[Flatten[covarianceOpp]]] // MatrixForm
```

$$\begin{pmatrix} 100 & 0 & 8 \\ 0 & 33 & 0 \\ 8 & 0 & 11 \end{pmatrix}$$

Now the distribution over the RGB-cube looks very different:

The samples cluster on the Goethe “Kantenfarben”, thus greens and purples are very rare.
(This is a view along the KW-axis, green on the left, purple on the right.)
We have treated this case in some detail in a previous publication (see text).

Again this apparent selectivity is due to a fully non-selective statistics.
The reason is that the spectum is not periodic, its two limits are more weakly correlated than any pair of wavelengths in the interior.

```
Graphics3D[
 {
  EdgeForm[],
  AbsolutePointSize[8],
  {
     Apply[RGBColor, #],
     Point[#]
    } & /@ rgbSamples,
  FaceForm[Red], Sphere[{1, 0, 0}, 0.05],
  FaceForm[Yellow], Sphere[{1, 1, 0}, 0.05],
  FaceForm[Green], Sphere[{0, 1, 0}, 0.05],
  FaceForm[Cyan], Sphere[{0, 1, 1}, 0.05],
  FaceForm[Blue], Sphere[{0, 0, 1}, 0.05],
  FaceForm[Magenta], Sphere[{1, 0, 1}, 0.05],
  FaceForm[White], Sphere[{1, 1, 1}, 0.05]
 },
 PlotRange → Table[{-0.1, 1.1}, {3}],
 Boxed → False,
 Lighting → "Neutral",
 Background → GrayLevel[0.7],
 ViewVertical → {0, 0.5, 1},
 ViewPoint → 1000 {1, 1, 1}
]
```

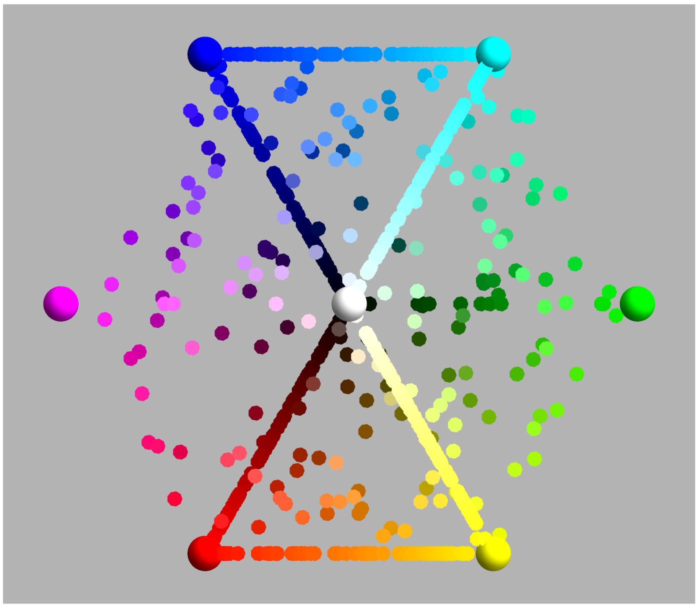

Supplement: Supplementary material [file SpectralCorrelation.pdf]
