# Supplementary material for: Colors of the Sublunar
Source: Iperception. 2017 Sep 29;8(5):2041669517733484. doi: 10.1177/2041669517733484 (PMC5624368; doi:10.1177/2041669517733484)

CIE Tables to RGB-representation

In this notebook you see how one starts from the CIE basic tables and in a few steps develops all the necessary structure for work in the RGB-representation.

Import data from the INTERNET

The first step is to import the tables from CIE site ("http://www.cie.co.at/index.php/LEFTMENUE/index.php?i_ca_id=298").
You obtain an Excel file, I import it here:

```
CIEData =
  Import["/Users/jankoenderink/Documents/MyWorkSpace/Manuscripts/Submitted/
     NaturalColorGamuts/AdditionalMaterial/CIE/CIE.xls"];
Length[CIEData]
```

```
5
```

At this point you might do things a little differently. You might like to work through Excel and MatLab. We use Mathematica because we’re used to that.
Thus we unpack the Excel data and turn the table into functions (so all necessary interpolation will be automatic) for us.

The CIEData contains 5 tables, of which we use only two.
The first is the average daylight spectrum :

```
CIEData[[2]][[3]][[1]]
```

```
Relative spectral power distribution of CIE Standard Illuminant D65
```

We turn it into an interpolation function, it is defined on the spectral range 305-830nm.
We use linear interpolation for simplicity (quadratic might be better, but cubic will introduce ringing).

```
d65 = Interpolation[Drop[Drop[CIEData[[2]], 6], -1], InterpolationOrder → 1]
```

```
InterpolatingFunction[ Domain: {{305., 830.}}
                       Output: scalar ]
```

Here is a plot:

```
Plot[
 d65[lambda],
 {lambda, 305, 830},
 PlotStyle → {Black, Thick},
 Filling → 0,
 Axes → None,
 Frame → True,
 FrameStyle → Table[{Black, Thin}, {4}],
 FrameTicksStyle → {Black, Thin},
 ImageSize → 500,
 BaseStyle → Directive[FontFamily → "Times", FontSize → 24],
 FrameTicks → {{{0, 50, 100}, None}, {Automatic, None}},
 FrameLabel → {{"radiant power", None}, {"wavelength (nm)", None}},
 PlotLabel → "CIE Illuminant D65"
]
```

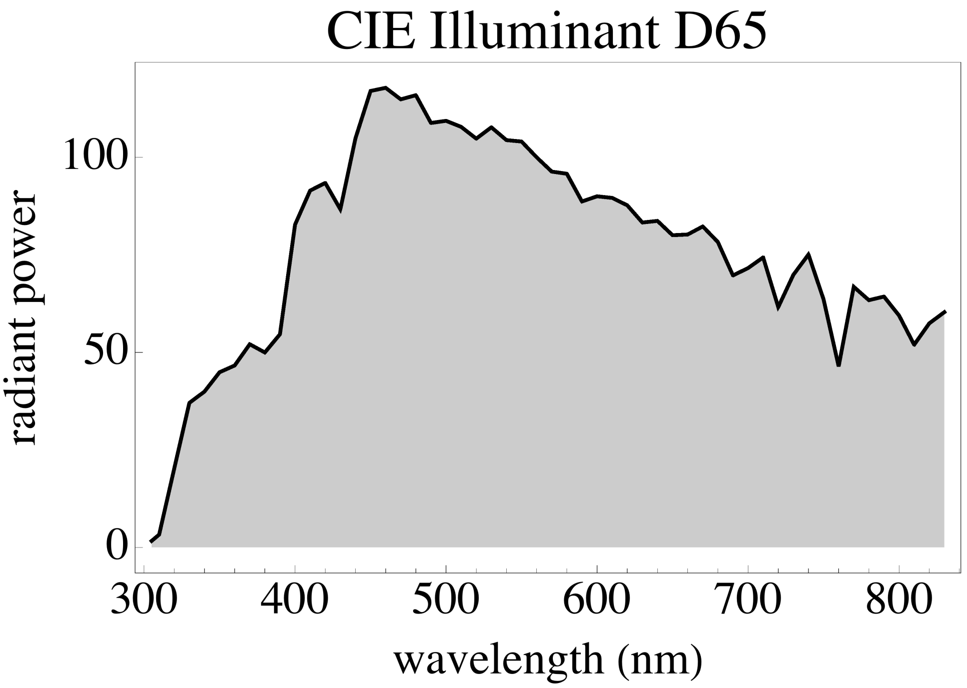

The second table involves the color matching functions.
Here we use the 1964 observer, you can do the same with the 1931 observer, of course.

```
CIEData[[5]][[2]][[1]]
```

```
CIE 1964 supplementary standard colorimetric observer
```

We turn it into a triple of interpolation functions, x, y and z:

```
Module[
  {dat, lambda, xDat, yDat, zDat},
  dat = Drop[Drop[CIEData[[5]], 5], -1];
  {lambda, xDat, yDat, zDat} = Transpose[dat];
  x = Interpolation[Transpose[{lambda, xDat}]];
  y = Interpolation[Transpose[{lambda, yDat}]];
  z = Interpolation[Transpose[{lambda, zDat}]];
 ];
```

Here the domain is different, it is 380-780nm:

```
{x, y, z}
```

```
{InterpolatingFunction[ Domain: {{380., 780.}} Output: scalar ],
 InterpolatingFunction[ Domain: {{380., 780.}} Output: scalar ],
 InterpolatingFunction[ Domain: {{380., 780.}} Output: scalar ]}
```

Putting the data in suitable form

We restrict the domain to 380-750nm, which is amply sufficient:

```
spectralLimits = {380, 750};
Plot[
 {x[lambda], y[lambda], z[lambda]},
 {lambda, spectralLimits[[1]], spectralLimits[[2]]},
 PlotRange → All,
 PlotStyle → {{Black, Thick}, {Blue, Thick}, {Orange, Thick}},
 Filling → 0,
 Axes → None,
 Frame → True,
 FrameStyle → Table[{Black, Thick}, {4}],
 FrameTicksStyle → {Black, Thick},
 BaseStyle → Directive[FontFamily → "Times", FontSize → 24],
 FrameTicks → {{{0, 1, 2}, None}, {{400, 500, 600, 700}, None}},
 FrameLabel → {{None, None}, {"wavelength (nm)", None}},
 PlotLabel → "CIE 1964 observer",
 ImageSize → 500
]
```

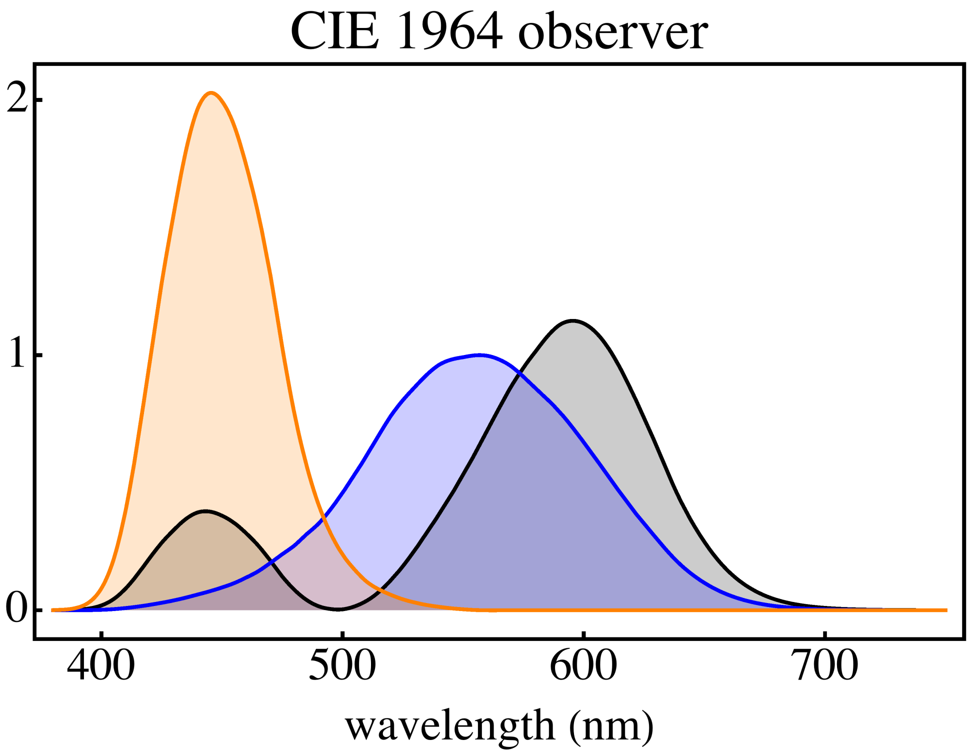

Daylight white

This is the daylight white:

```
white = NIntegrate[d65[lambda] {x[lambda], y[lambda], z[lambda]},
  {lambda, spectralLimits[[1]], spectralLimits[[2]]}]
```

```
{11017., 11620.1, 12468.8}
```

You may like the normalized form better:

```
white / Apply[Plus, white]
```

```
{0.313822, 0.331001, 0.355177}
```

Finding the cut-loci for the optimal RGB-representation (the Schopenhauer “parts of daylight”)

The next task is to find the optimal spectral cuts. To find this we first find the volume of the crate spanned by the colors for an arbirary cut:

```
spannedVolume[firstCut_, secondCut_] :=
 Det[
  {
   NIntegrate[d65[w] {x[w], y[w], z[w]}, {w, firstCut, secondCut}],
   NIntegrate[d65[w] {x[w], y[w], z[w]}, {w, spectralLimits[[1]], firstCut}],
   NIntegrate[d65[w] {x[w], y[w], z[w]}, {w, secondCut, spectralLimits[[2]]}]
  }
 ]
```

Since we roughly know where the cuts will be (try for yourself), we search only in a narrow environment.
This takes some time.

```
{maxVol, sol} =
 Maximize[
  {spannedVolume[w1, w2], 470 < w1 < 490, 560 < w2 < 575},
  {w1, w2}
 ]
```

$\{3.93666\times 10^{11}, \{w1 \to 482.65, w2 \to 565.433\}\}$

Here is the solution Mathematica found in a few minutes:

```
{cutL, cutH} = {w1, w2} /. sol
```

```
{482.65, 565.433}
```

The cube root of the maxium volume is

```
maxVol ^ (1 / 3)
```

```
7328.97
```

Which is comparable to the (very rough and an upper limit) estimate we get from the white point:

```
Norm[white] / Sqrt[3]
```

```
11717.1
```

Now we may plot the “parts of daylight” in the spectral representation:

```
Graphics[
 {
  LightGray,
  Rectangle[{305, 0}, {830, 120}],
  Blue,
  Polygon[Join[{{305, 0}}, Table[{w, d65[w]}, {w, 305, cutL, 0.1}], {{cutL, 0}}]],
  Green, Polygon[Join[{{cutL, 0}},
    Table[{w, d65[w]}, {w, cutL, cutH, 0.1}], {{cutH, 0}}]],
  Red, Polygon[Join[{{cutH, 0}}, Table[{w, d65[w]}, {w, cutH, 830, 0.1}],
    {{830, 0}}]],
  Black, AbsoluteThickness[2], Line[Table[{w, d65[w]}, {w, 305, 830}]]
 },
 PlotRange → {{380, 710}, {0, 120}},
 PlotRangeClipping → True,
 Axes → None,
 Frame → True,
 FrameStyle → Table[{Black, Thick}, {4}],
 FrameTicksStyle → {Black, Thick},
 ImageSize → 500,
 BaseStyle → Directive[FontFamily → "Times", FontSize → 24],
 FrameTicks → {{{0, 50, 100}, None}, {{400, 500, 600, 700}, None}},
 FrameLabel → {{"radiant power", None}, {"wavelength (nm)", None}},
 PlotLabel → "CIE Illuminant D65",
 AspectRatio → 1 / GoldenRatio
]
```

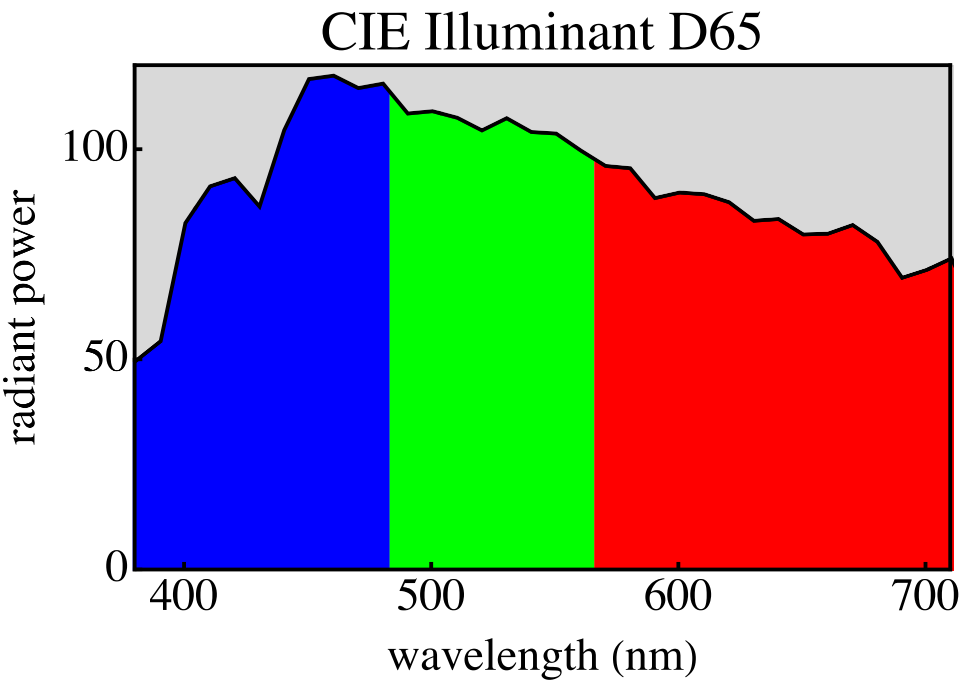

The RGB “parts of daylight”

This allows us to compute the colors of the parts:

```
blue = NIntegrate[d65[w] {x[w], y[w], z[w]}, {w, spectralLimits[[1]], cutL}];
green = NIntegrate[d65[w] {x[w], y[w], z[w]}, {w, cutL, cutH}];
red = NIntegrate[d65[w] {x[w], y[w], z[w]}, {w, cutH, spectralLimits[[2]]}];
```

A sanity check:

```
red + green + blue == white
True
```

The RGB-display gamut

This is the RGB-triangle in the CIE xy-chromaticity diagram:

```
xyL = Map[Append[Drop[# / Apply[Plus, #], -1], #[[2]]] &, {red, green, blue}]
{{0.608388, 0.391612, 4390.3},
 {0.221856, 0.663213, 6413.66}, {0.144189, 0.0573749, 816.129}}
```

The white point:

```
Append[Drop[white / Apply[Plus, white], -1], white[[2]]]
{0.313822, 0.331001, 11 620.1}
```

The ratio of luminances:

```
100 Map[#[[2]] &, {red, green, blue}] / white[[2]] // Round
{38, 55, 7}
```

The gamut is very similar to what one gets for typical displat units:

```
Graphics[
 {
  EdgeForm[{Black, AbsoluteThickness[1]}],
  FaceForm[Gray],
  Polygon[Table[{x[w], y[w]} / (x[w] + y[w] + z[w]),
    {w, spectralLimits[[1]], spectralLimits[[2]], 1}]],
  FaceForm[LightGray],
  Polygon[{Drop[xyL[[1]], -1], Drop[xyL[[2]], -1], Drop[xyL[[3]], -1]}],
  EdgeForm[{Black, AbsoluteThickness[1]}],
  FaceForm[Red], Disk[Drop[xyL[[1]], -1], 0.02],
  FaceForm[Green], Disk[Drop[xyL[[2]], -1], 0.02],
  FaceForm[Blue], Disk[Drop[xyL[[3]], -1], 0.02],
  FaceForm[White], Disk[{1, 1} / 3, 0.02]
 }
]
```

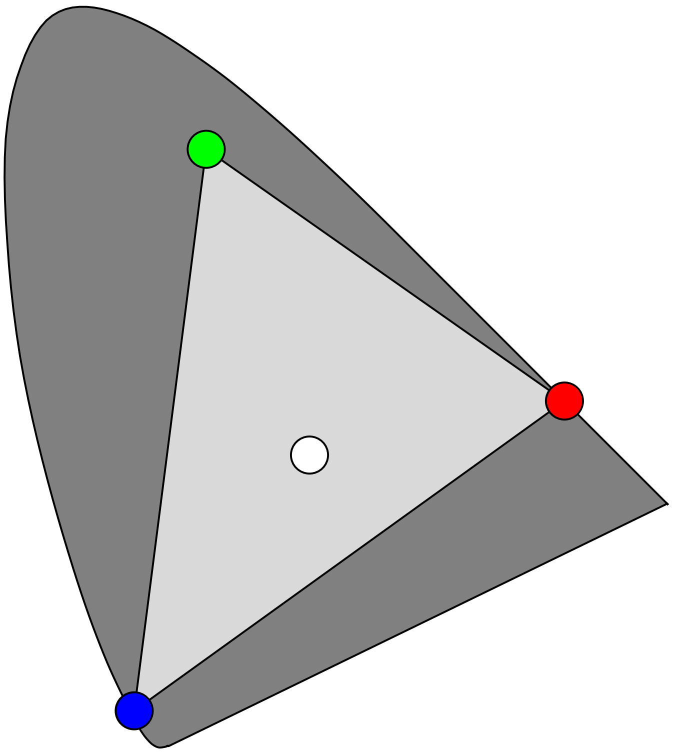

The chromaticity diagram is NOT a good way to judge quality!

Many display units appear to “do better in the red”, but that implies that they actually claim a smaller volume in color space.
Most modern units are “good enough” though.

The RGB color matching functions

Since we want to express all colors in terms of the red, green and blue parts of daylight, we need a transformation matrix:

```
matrix = Inverse[Transpose[{red, green, blue}]];
MatrixForm[matrix]
```

$$\begin{pmatrix} 0.000182731 & -0.0000561067 & -0.0000289675 \\ -0.000126661 & 0.000196774 & 8.73362\times 10^{-6} \\ 0.0000123953 & -0.0000192567 & 0.000087194 \end{pmatrix}$$

The parts of daylight generate the standard RGB cardinal colors:

```
cyan = green + blue;
magenta = blue + red;
yellow = red + green;
white = red + green + blue;
black = {0, 0, 0};
```

```
{matrix.red, matrix.green, matrix.blue} // Chop // MatrixForm
```

$$\begin{pmatrix} 1. & 0 & 0 \\ 0 & 1. & 0 \\ 0 & 0 & 1. \end{pmatrix}$$

```
{matrix.cyan, matrix.magenta, matrix.yellow} // Chop // MatrixForm
```

$$\begin{pmatrix} 0 & 1. & 1. \\ 1. & 0 & 1. \\ 1. & 1. & 0 \end{pmatrix}$$

Here are the RGB color matching curves:

```
Module[
 {r, g, b, wav, rgb},
 rgb = Table[matrix.{x[w], y[w], z[w]},
   {w, spectralLimits[[1]], spectralLimits[[2]], 1}];
 rgb /= Max[Flatten[rgb]];
 {r, g, b} = Transpose[rgb];
 wav = Table[w, {w, spectralLimits[[1]], spectralLimits[[2]], 1}];
 r = Transpose[{wav, r}];
 g = Transpose[{wav, g}];
 b = Transpose[{wav, b}];
 ListPlot[
  {r, g, b},
  Joined → True,
  PlotRange → {{380, 710}, All},
  PlotStyle → {{Red, Thick}, {RGBColor[0, 0.7, 0], Thick}, {Blue, Thick}},
  Filling → 0,
  Axes → None,
  Frame → True,
  FrameStyle → Table[{Black, Thick}, {4}],
  FrameTicksStyle → {Black, Thick},
  BaseStyle → Directive[FontFamily → "Times", FontSize → 24],
  FrameTicks → {{{0, 0.5, 1}, None}, {{400, 500, 600, 700}, None}},
  FrameLabel → {{None, None}, {"wavelength (nm)", None}},
  PlotLabel → "RGB color matching curves",
  Prolog → {LightGray, Rectangle[{380, -0.25}, {710, 110}]},
  Epilog → {Black, Thick, Line[{{380, 0}, {710, 0}}]},
  ImageSize → 500
 ]
]
```

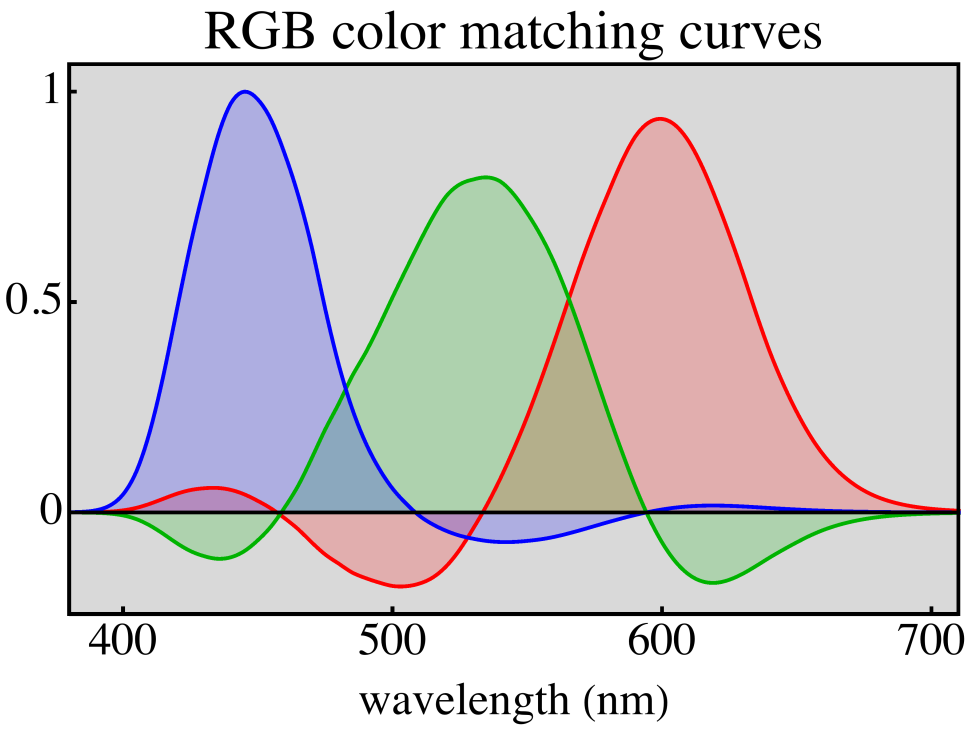

They are mutually lightly correlated:

```
cv = Covariance[Table[matrix.{x[w], y[w], z[w]},
     {w, spectralLimits[[1]], spectralLimits[[2]], 1}]];
cv /= Max[Flatten[cv]];
MatrixForm[Round[100 cv]]
```

$$\begin{pmatrix} 100 & -22 & -24 \\ -22 & 86 & -26 \\ -24 & -26 & 89 \end{pmatrix}$$

The Goethe edge colors (Kantenfarben)

The “edge colors” are simply the low and high pass spectra:

```
edgecolors =
  Module[
   {delta, n = 74},
   delta = (spectralLimits[[2]] - spectralLimits[[1]]) / n;
   Table[
    matrix.NIntegrate[d65[w] {x[w], y[w], z[w]}, {w, spectralLimits[[1]], v}],
    {v, spectralLimits[[1]], spectralLimits[[2]], delta}
   ]
  ];
```

```
Graphics3D[
 {
  AbsolutePointSize[12],
  Map[{Apply[RGBColor, #], Point[#]} &, edgecolors],
  Map[{Apply[RGBColor, {1, 1, 1} - #], Point[{1, 1, 1} - #]} &, edgecolors]
 },
 Axes → True,
 AxesLabel → {"R", "G", "B"}
]
```

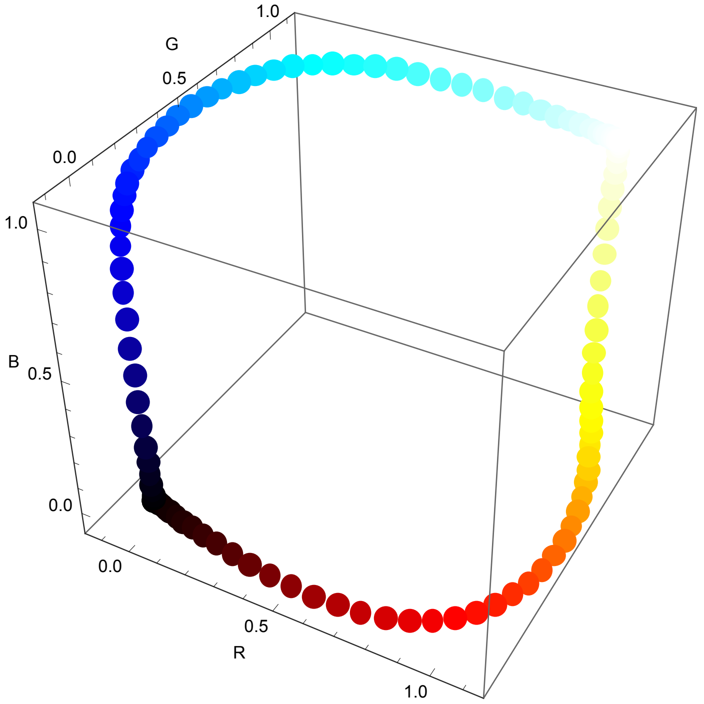

The edge colors lie on the “seams” of the color solid (next).

The Schrödinger optimal colors

Schrödinger’s optimal colors are simply slices of daylight. We compute a table, which takes some time, so we simple store the table once and for all.
The table is really redundant, but so what, memory and disk space are cheap. (THIS TAKES TIME!)

```
optimalColors =
  Module[
   {delta, n = 74},
   delta = (spectralLimits[[2]] - spectralLimits[[1]]) / n;
   Table[
    Table[
     Which[
      v1 < v2, matrix.NIntegrate[d65[w] {x[w], y[w], z[w]}, {w, v1, v2}],
      v1 > v2, matrix.(white - NIntegrate[d65[w] {x[w], y[w], z[w]}, {w, v2, v1}]),
      True, {0, 0, 0}
     ],
     {v2, spectralLimits[[1]], spectralLimits[[2]], delta}
    ],
    {v1, spectralLimits[[1]], spectralLimits[[2]], delta}
   ]
  ];
```

```
optimalColors // Dimensions
```

```
{75, 75, 3}
```

```
n = First[Dimensions[optimalColors]]
```

```
75
```

Now for some graphics. Here is the RGB-crate:

```
crate =
  {
   EdgeForm[],
   FaceForm[{White, Specularity[White, 50]}],
   Cylinder[{{0, 0, 0}, {1, 0, 0}}, 0.02],
   Cylinder[{{0, 0, 0}, {0, 1, 0}}, 0.02],
   Cylinder[{{0, 0, 0}, {0, 0, 1}}, 0.02],
   Cylinder[{{1, 1, 1}, {1, 1, 0}}, 0.02],
   Cylinder[{{1, 1, 1}, {0, 1, 1}}, 0.02],
   Cylinder[{{1, 1, 1}, {1, 0, 1}}, 0.02],
   Tube[{{1, 1, 0}, {0, 1, 0},
     {0, 1, 1}, {0, 0, 1}, {1, 0, 1}, {1, 0, 0}, {1, 1, 0}}, 0.02],
   FaceForm[{Black, Specularity[White, 50]}],
   Cylinder[{{0, 0, 0}, {1, 1, 1}}, 0.01],
   FaceForm[{Black,   Specularity[White, 50]}], Sphere[{0, 0, 0}, 0.05],
   FaceForm[{White,   Specularity[White, 50]}], Sphere[{1, 1, 1}, 0.05],
   FaceForm[{Red,     Specularity[White, 50]}], Sphere[{1, 0, 0}, 0.05],
   FaceForm[{Green,   Specularity[White, 50]}], Sphere[{0, 1, 0}, 0.05],
   FaceForm[{Blue,    Specularity[White, 50]}], Sphere[{0, 0, 1}, 0.05],
   FaceForm[{Cyan,    Specularity[White, 50]}], Sphere[{0, 1, 1}, 0.05],
   FaceForm[{Magenta, Specularity[White, 50]}], Sphere[{1, 0, 1}, 0.05],
   FaceForm[{Yellow,  Specularity[White, 50]}], Sphere[{1, 1, 0}, 0.05]
  };
crateGraphics = Graphics3D[crate, Boxed → False]
```

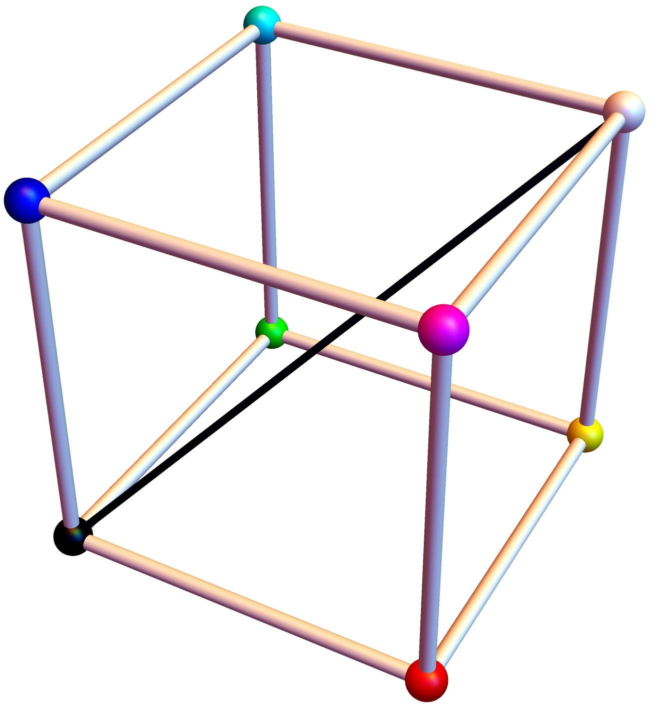

The color solid

We compute the surface of the color solid in two parts: the bandpass and the bandgap optimal colors.

```
bandpassColors =
  {
   EdgeForm[],
   Table[
    Table[
     {
      FaceForm[{Apply[RGBColor, optimalColors[[i, j]]], Opacity[1]}],
      Polygon[
       {
        optimalColors[[i, j]],
        optimalColors[[i + 1, j]],
        optimalColors[[i + 1, j + 1]],
        optimalColors[[i, j + 1]]
       }
      ]
     },
     {j, i, n - 1}
    ],
    {i, 1, n - 2}
   ]
  };
bandpassGraphics = Graphics3D[
 bandpassColors,
  Lighting → "Neutral",
  Boxed → False
 ]
```

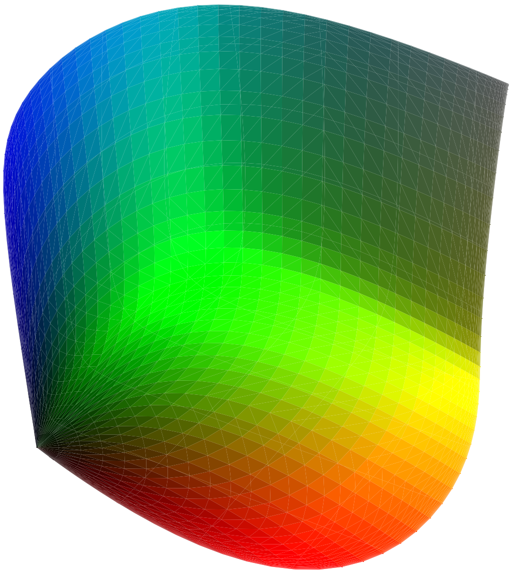

```
Show[{crateGraphics, bandpassGraphics}]
```

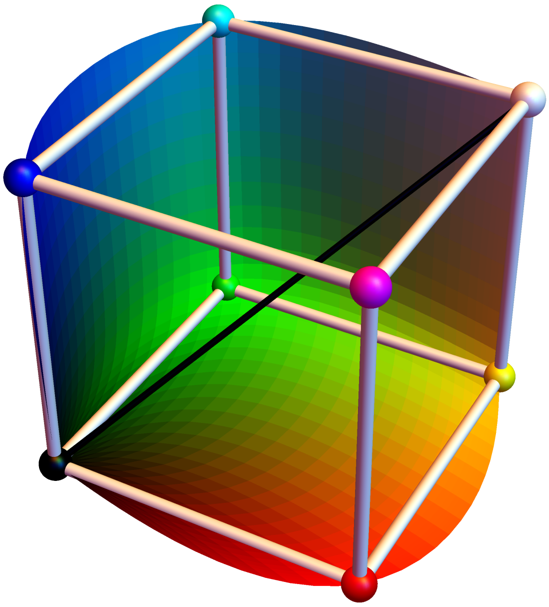

```
bandgapColors =
  {
   Table[
    Table[
     {
      FaceForm[{Apply[RGBColor, {1, 1, 1} - optimalColors[[i, j]]], Opacity[1]}],
      Polygon[
       {
        {1, 1, 1} - optimalColors[[i, j]],
        {1, 1, 1} - optimalColors[[i + 1, j]],
        {1, 1, 1} - optimalColors[[i + 1, j + 1]],
        {1, 1, 1} - optimalColors[[i, j + 1]]
       }
      ]
     },
     {j, i, n - 1}
    ],
    {i, 1, n - 2}
   ]
  };
bandgapGraphics = Graphics3D[
  bandgapColors,
  Lighting → "Neutral",
  Boxed → False
 ]
```

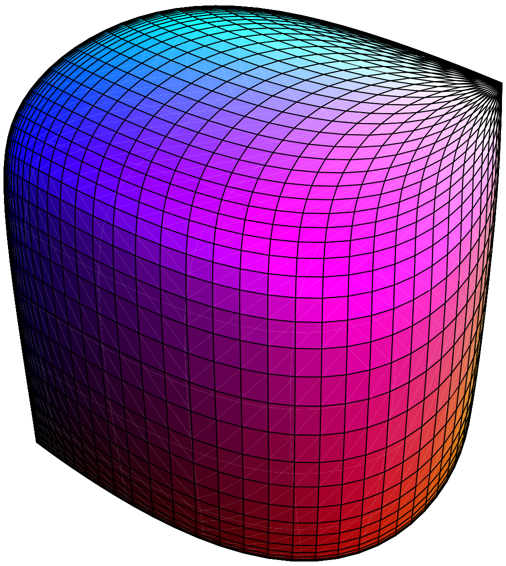

```
Show[{crateGraphics, bandgapGraphics}]
```

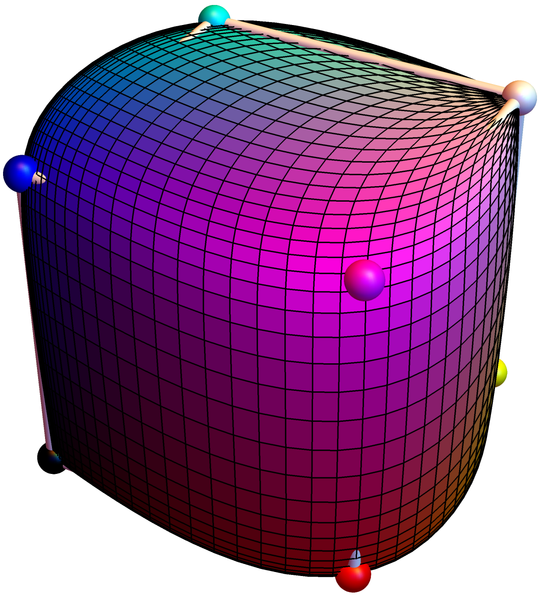

Here is a picture of the Schrödinger color solid. The RGB crate lies in its interior.

```
Show[{bandpassGraphics, bandgapGraphics}]
```

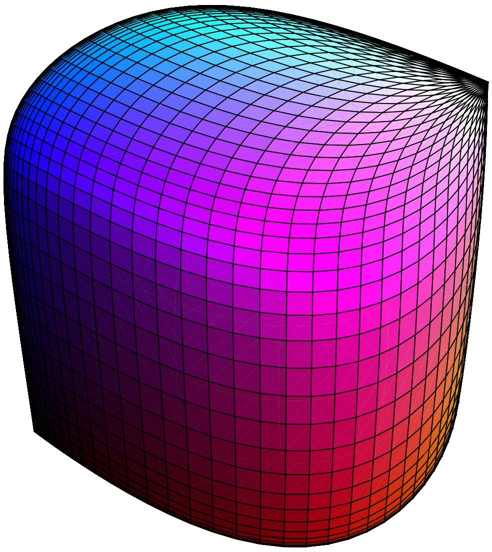

Supplement: Supplementary material [file CIE.pdf]
