# Supplementary material for: Colors of the Sublunar
Source: Iperception. 2017 Sep 29;8(5):2041669517733484. doi: 10.1177/2041669517733484 (PMC5624368; doi:10.1177/2041669517733484)

Photographic imaging

In photographic imaging the physical radiance is mapped on a standard “intensity” (say) domain.

The radiance is a non-negative quantity, it lives on (0,∞), whereas the intensity lives on some finite interval, here we use [0,1] (in practice it will often be [0,255], this makes no essential difference).

Define a scene (radiances on (0,∞))

We need an example.
Here we use an arbitrarily chosen image (that makes it easier to judge results, it is fully irrelevant what one uses here---but if we used a random noise image no one would be able to make sense of the results) to simulate an arbitrary radiance distribution, just for kicks.

Radiance field (no need to know how we did it)

Histogram

The radiance is non - negative:

```
{Min[Flatten[radiances]], Max[Flatten[radiances]]}
```

```
{0.147022, 1.7799}
```

(Of course the histogram is irrelevant, it is just an example. Anyway, all values are positive!)

```
Histogram[Flatten[radiances]]
```

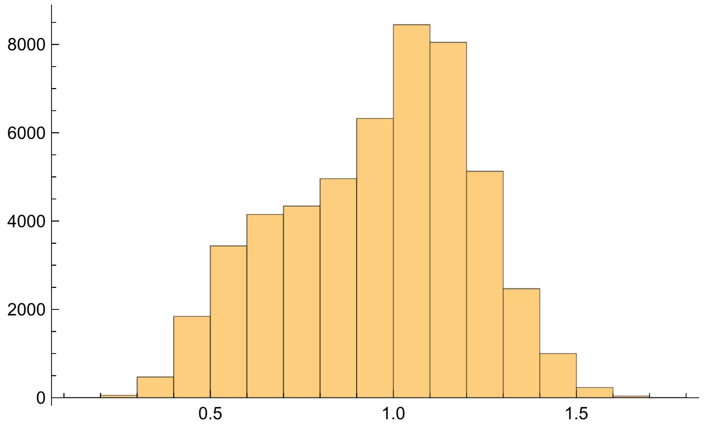

Map on the log-radiance domain (on (-∞,+∞))

The default (Jeffreys) prior for non-negative quantities is hyperbolic, thus the natural domain is the log-domain.

```
logRadiances = Log[10, radiances];
{Min[Flatten[logRadiances]], Max[Flatten[logRadiances]]}
```

```
{-0.832618, 0.250396}
```

The log-radiances are distributed over (-∞,+∞):

```
Histogram[Flatten[logRadiances]]
```

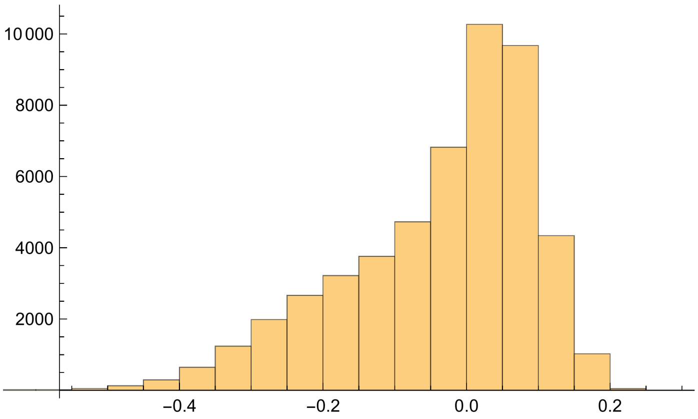

Define compressing ogive (maps [-∞,+∞]) on (0,1))

The imaging process implements a compression, mapping (-∞,+∞) on (0,1).
We model it with some ogive function omega (any will do)

```
omega[x_] := (1 + Tanh[x]) / 2;
Plot[omega[x], {x, -4, 4}, PlotStyle → {Thick, Blue}, Filling → Axis]
```

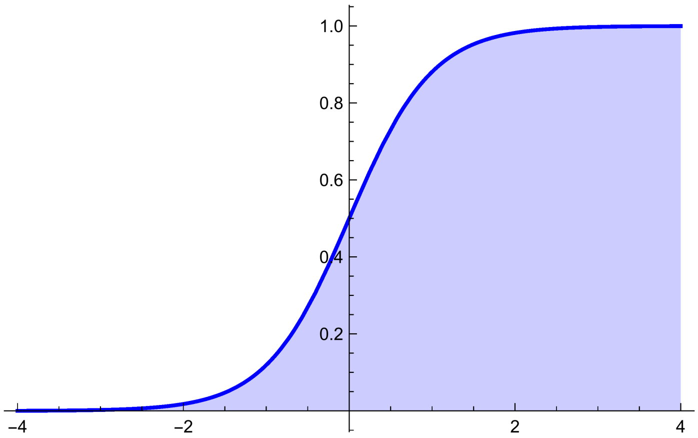

Examples

Now we may start imaging!
The parameters are the “exposure”, the “contrast range” and the “gamma”.

Here we shift omega, this is what “exposure” does:

```
Plot[Evaluate[Table[omega[x - x0], {x0, -2, 2}]], {x, -4, 4}, PlotStyle → Thick]
```

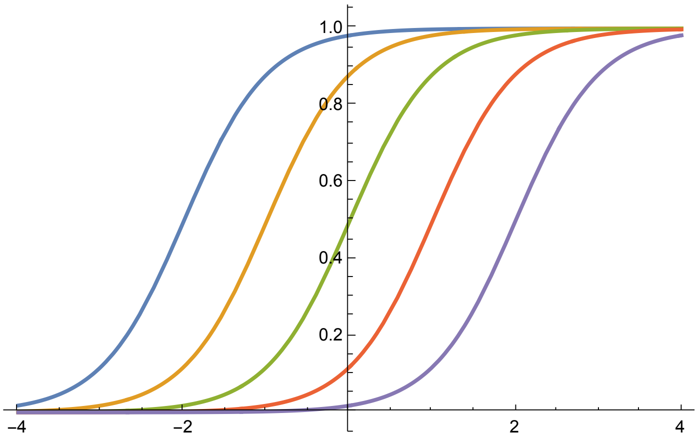

Here we stretch omega, this is what “contrastRange” does:

```
Plot[Evaluate[Table[omega[x / 2^i], {i, -2, 2}]], {x, -4, 4}, PlotStyle → Thick]
```

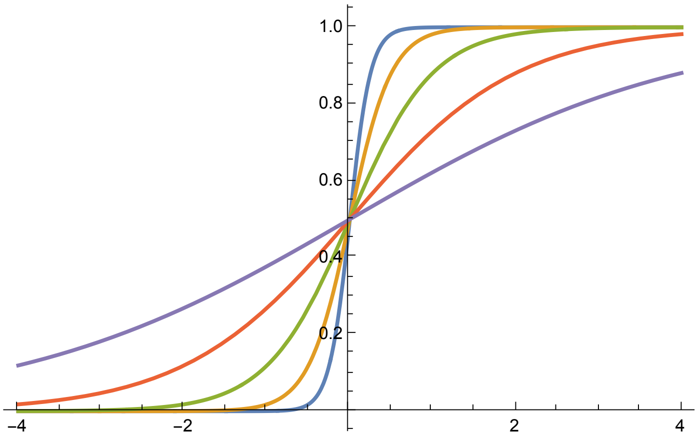

For a start we ignore the gamma-transformation.

A

Notice: “exposure” is WHERE we apply omega, whereas “contrastRange” is how much we stretch omega.
Here is the first example:

```
exposure = -0.1;
contrastRange = 0.2;
```

```
mappedLogRadiance =
  Map[omega[(# - exposure) / contrastRange] &, logRadiances, {2}];
{Min[Flatten[mappedLogRadiance]], Max[Flatten[mappedLogRadiance]]}
```

```
{0.000657653, 0.9708}
```

The intensites are limited to (0,1):

```
Histogram[Flatten[mappedLogRadiance]]
```

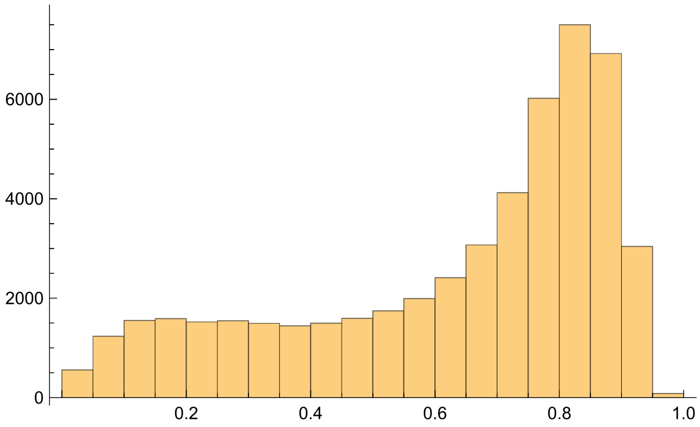

Here is the image:

```
Image[mappedLogRadiance]
```

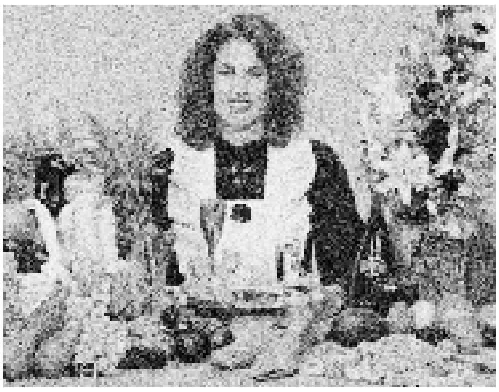

B

Here is another shot, changing the exposure and the contrast range:

```
exposure = -0.2;
contrastRange = 0.1;

mappedLogRadiance =
  Map[omega[(# - exposure) / contrastRange] &, logRadiances, {2}];
{Min[Flatten[mappedLogRadiance]], Max[Flatten[mappedLogRadiance]]}
```

$\{3.20002 \times 10^{-6}, 0.999878\}$

```
Histogram[Flatten[mappedLogRadiance]]
```

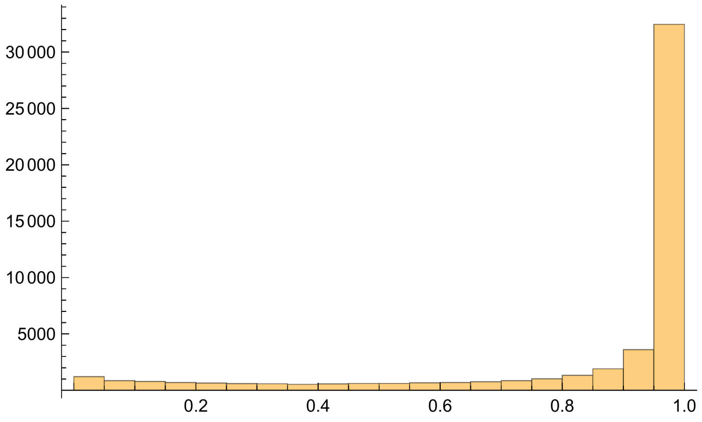


```
Image[mappedLogRadiance]
```

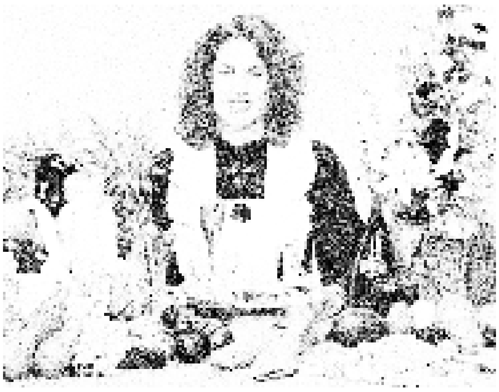

C

Yet another shot:

```
exposure = 0;
contrastRange = 0.4;
```

```
mappedLogRadiance =
  Map[omega[(# - exposure) / contrastRange] &, logRadiances, {2}];
{Min[Flatten[mappedLogRadiance]], Max[Flatten[mappedLogRadiance]]}
```

```
{0.0153211, 0.777642}
```

```
Histogram[Flatten[mappedLogRadiance]]
```

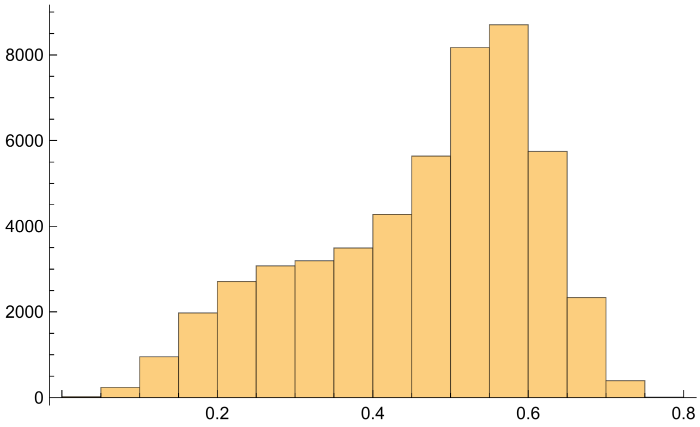


```
Image[mappedLogRadiance]
```

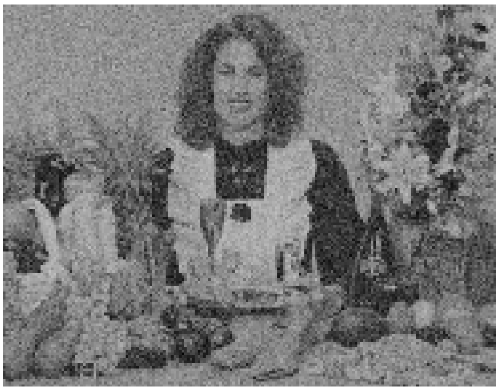

D

Apparently exposure and contrast range yield a lot of freedom.
Now we introduce a gamma-transformation.

This is what gamma transformation does:

```
Plot[Evaluate[Table[x^(2^i), {i, -2, 2}]], {x, 0, 1},
 AspectRatio → Automatic, AxesLabel → {"input intensity", "output intensity"}]
```

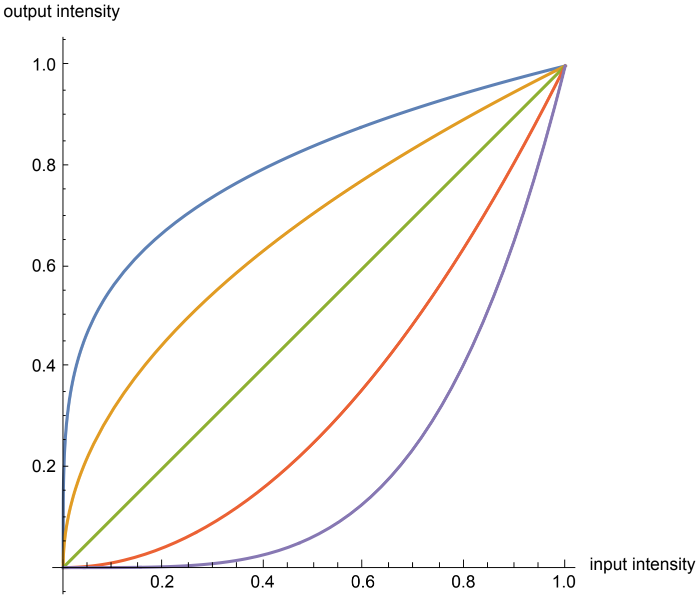

We can also show it as a change of the omega function:

```
Plot[Evaluate[Table[omega[x]^(2^i), {i, -2, 2}]], {x, -4, 4}, PlotStyle → Thick]
```

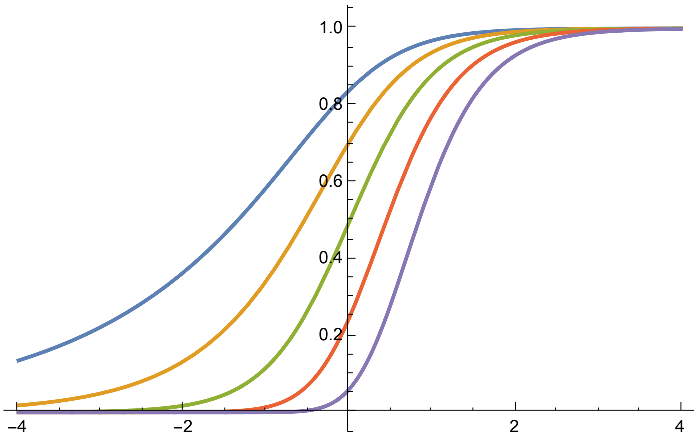

A gamma is very similar to some combined exposure-contrastRange change.
We show it mainly because so common.

```
exposure = 0;
contrastRange = 0.4;
```

```
gamma = 1.5;
```

```
mappedLogRadiance =
  Map[(omega[(# - exposure) / contrastRange])^gamma &, logRadiances, {2}];
{Min[Flatten[mappedLogRadiance]], Max[Flatten[mappedLogRadiance]]}
```

```
{0.00189641, 0.685756}
```

```
Histogram[Flatten[mappedLogRadiance]]
```

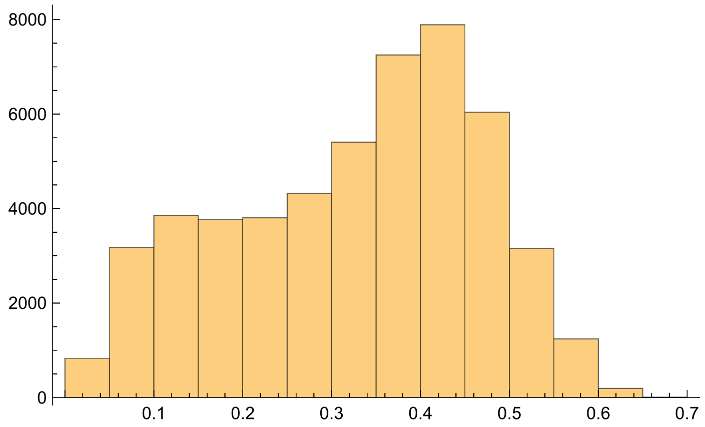


```
Image[mappedLogRadiance]
```

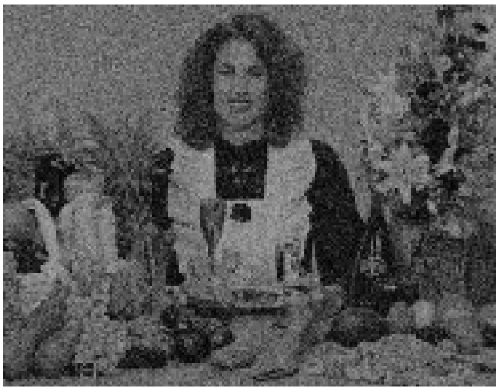

E

Here is the same exposure 0, the same contrast range, but a different gamma:

```
exposure = 0;
contrastRange = 0.4;

gamma = 0.5;

mappedLogRadiance =
  Map[(omega[(# - exposure) / contrastRange])^gamma &, logRadiances, {2}];
{Min[Flatten[mappedLogRadiance]], Max[Flatten[mappedLogRadiance]]}
```

```
{0.123778, 0.88184}
```

```
Histogram[Flatten[mappedLogRadiance]]
```

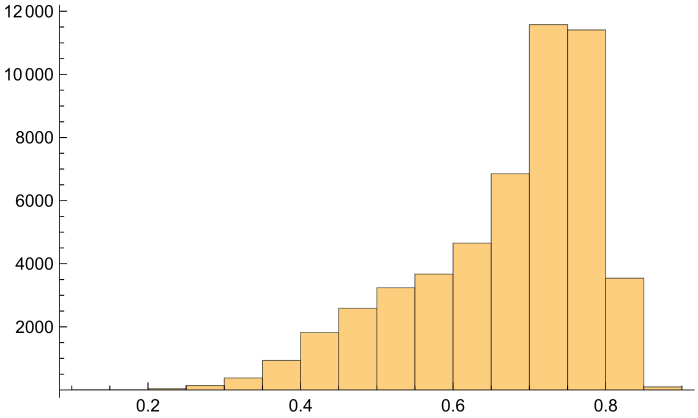


```
Image[mappedLogRadiance]
```

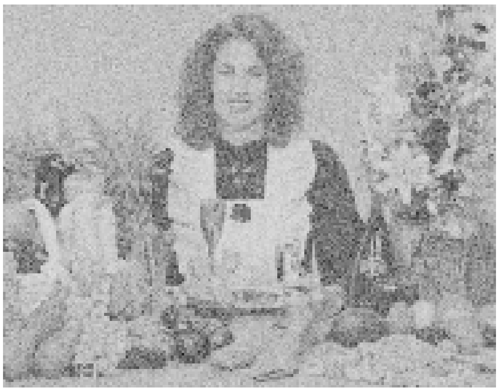

Supplement: Supplementary material [file Imaging.pdf]
