# Supplementary material for: Colors of the Sublunar
Source: Iperception. 2017 Sep 29;8(5):2041669517733484. doi: 10.1177/2041669517733484 (PMC5624368; doi:10.1177/2041669517733484)
Supplement: Supplementary material [file Sand.pdf]

Sand (example for small, single image)

This is an example for a small image, so the computations are fast. For large images or databases of images the computations may take several minutes.

Import an image and set up the database

Import an image (at this point one might want to do various additional things, like stripping off a transparance channel, cropping, and so forth)

```
image = Import[
  "/Users/jankoenderink/Documents/MyWorkSpace/Manuscripts/WorkingOnIt/
    NaturalColorGamuts/ImageDatabases/Sand.jpg"]
```

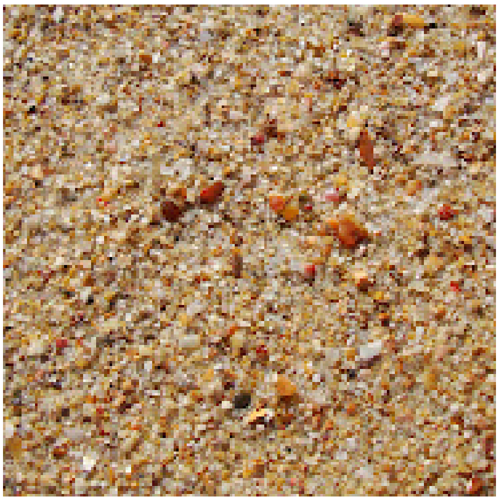

The data volume is smallish:

```
ImageDimensions[image]
```

```
{256, 256}
```

```
dataVolume = Apply[Times, ImageDimensions[image]]
```

```
65 536
```

Set up the database as a flat map of spectra:

```
rgb = Flatten[ImageData[image], 1];
```

```
Dimensions[rgb]
```

```
{65 536, 3}
```

```
rgb[[RandomInteger[65 536]]]
```

```
{0.647059, 0.490196, 0.34902}
```

The overall color is brownish:

```
meanRGB = Mean[rgb]
```

```
{0.673699, 0.538737, 0.388524}
```

```
Graphics[{Apply[RGBColor, meanRGB], Rectangle[{0, 0}, {1, 1}]}, ImageSize → 256]
```

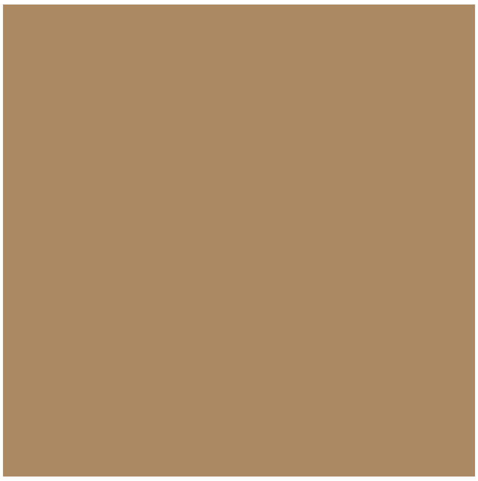

Randomly sampling from the database reveals a wide range of colors though:

```
sampleFomRGBDatabase[tileSize_, numTiles_] :=
 Graphics[
  {
   EdgeForm[],
   Table[
    {
     Apply[RGBColor, rgb[[RandomInteger[{1, dataVolume}]]]],
     Rectangle[{i - 1, j - 1}, {i, j}]
    },
    {i, 1, numTiles}, {j, 1, numTiles}
   ]
  },
  ImageSize → 256
 ]
```

```
sampleFomRGBDatabase[16, 32]
```

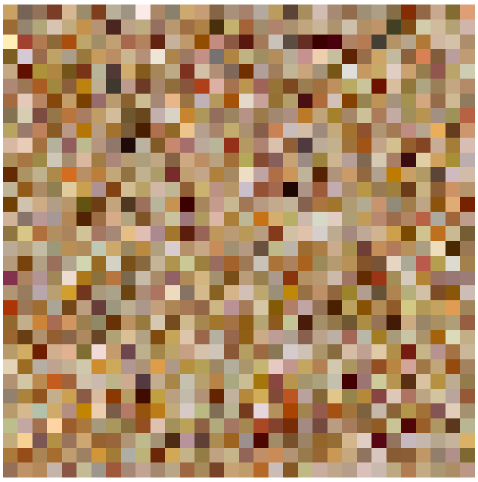

Compare this with the mean image!

Let the data speak

RGB histograms

The R, G and B histograms look rather nice, except for some debris near zero

```
SmoothHistogram[
 Transpose[rgb],
 ImageSize → 500,
 PlotStyle → {{Red, Thick}, {Green, Thick}, {Blue, Thick}},
 Filling → 0,
 PlotRange → {{0, 1}, All},
 PlotRangeClipping → True,
 Axes → None,
 Frame → True,
 FrameStyle → Table[{Black, Thin}, {4}],
 FrameTicksStyle → {Black, Thin},
 BaseStyle → Directive[FontFamily → "Times", FontSize → 24],
 PlotLabel → "RGB histograms"
]
```

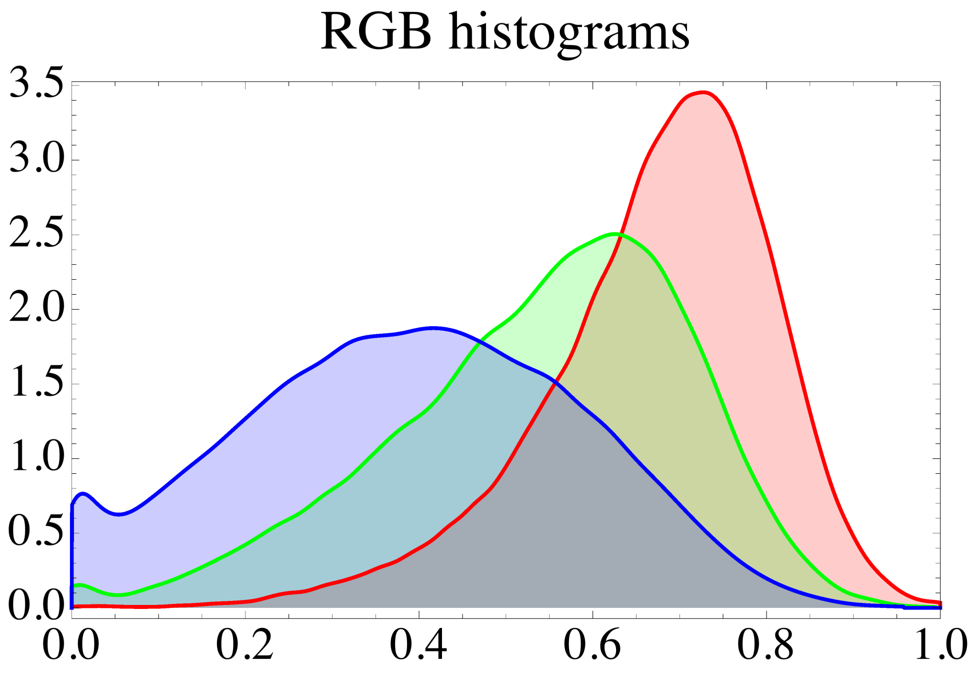

RGB covariance structure

The covariance of the RGB channels:

```
CRGB = Covariance[rgb];
Round[100 CRGB / Max[Flatten[CRGB]]] // MatrixForm
```

$$\begin{pmatrix} 47 & 55 & 52 \\ 55 & 79 & 80 \\ 52 & 80 & 100 \end{pmatrix}$$

Map to physical space (RGB to $\rho\chi\beta$)

The ogive function

Define the ogive transfer function and its inverse

```
g[y_] := ArcTanh[2 y - 1];
```

```
Plot[g[y], {y, -0.3, 1.3}, PlotLabel → "g[y]"]
```

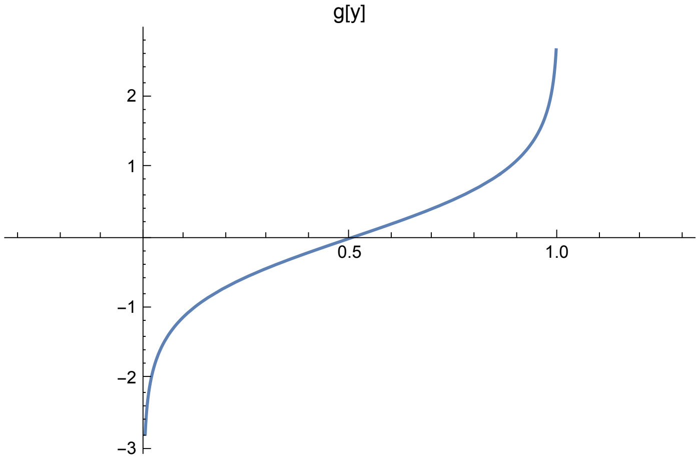

Map to physical space

Remove debris accumulated near the ends of the RGB values scale (here 0-1, in many applications 0-255)

```
upperCutoff = 0.95;
bottomCutoff = 0.05;
```

```
rgb = Select[rgb,
   (
     (bottomCutoff < #[[1]] < upperCutoff) &&
      (bottomCutoff < #[[2]] < upperCutoff) &&
      (bottomCutoff < #[[3]] < upperCutoff)
    ) &];
(dataVolume - Length[rgb]) / dataVolume // N
```

```
0.0525665
```

So we lost a small fraction of the data, the data volume becomes:

```
dataVolume = Length[rgb]
```

```
62 091
```

Here we do the actual transform. Notice that the histograms become more nearly normal:

```
ρ = Map[g, Map[#[[1]] &, rgb]];
χ = Map[g, Map[#[[2]] &, rgb]];
β = Map[g, Map[#[[3]] &, rgb]];
```

```
SmoothHistogram[
 {ρ, χ, β},
 PlotRange → {{g[bottomCutoff], g[upperCutoff]}, All},
 ImageSize → 500,
 PlotStyle → {{Red, Thick}, {Green, Thick}, {Blue, Thick}},
 Filling → 0,
 PlotRangeClipping → True,
 Axes → None,
 Frame → True,
 FrameStyle → Table[{Black, Thin}, {4}],
 FrameTicksStyle → {Black, Thin},
 BaseStyle → Directive[FontFamily → "Times", FontSize → 24],
 PlotLabel → "ρχβ histograms"
]
```

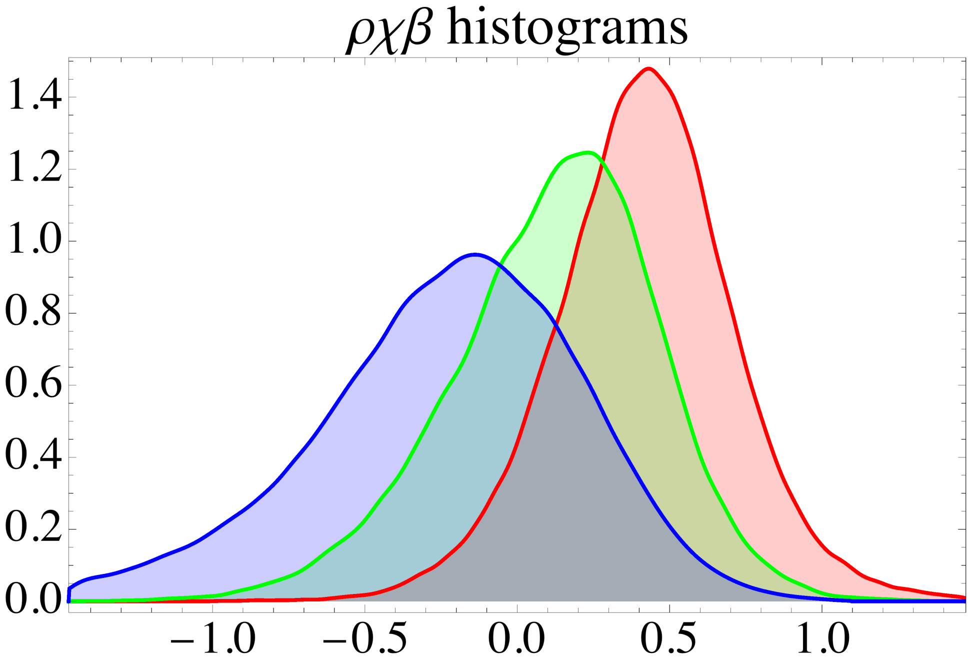

ρχβ covariance in the physical domain

The rgb-covariance in the physical domain

```
Cρχβ = Covariance[Transpose[{ρ, χ, β}]];
Round[100 Cρχβ / Max[Flatten[Cρχβ]]] // MatrixForm
```

$$\begin{pmatrix} 47 & 49 & 50 \\ 49 & 63 & 70 \\ 50 & 70 & 100 \end{pmatrix}$$

```
Eigenvalues[Cρχβ] / Apply[Plus, Eigenvalues[Cρχβ]]
```

{0.896781, 0.0859609, 0.0172577}

```
Round[100 Map[# / Max[Abs[#]] &, Eigenvectors[Cρχβ ]]] // MatrixForm
```

63	81	100
-100	-34	91
68	-100	37

Λ, Θ, X (canonical opponent system in the physical domain, ρχβ to ΛΘX)

Here we do the standard transformation to the canonical opponent basis:

```
matrixT =
  N[{
     {4, 4, 4},
     {6, 0, -6},
     {-3, 6, -3}
    } / 12
  ];
MatrixForm[matrixT]
```

0.333333	0.333333	0.333333
0.5	0.	-0.5
-0.25	0.5	-0.25

```
ΛΘX = Map[matrixT.# &, Transpose[{ρ, χ, β}]];
```

We compute the covariance matrix

```
CΛΘX   = Covariance[ΛΘX];
Round[100 CΛΘX / Max[Flatten[CΛΘX]]] // MatrixForm
```

100	-20	0
-20	19	2
0	2	3

```
CΘX = Drop[Map[Drop[#, 1] &, CΛΘX], 1];
Round[100 CΘX / Max[Flatten[ CΘX]]] // MatrixForm
```

100	10
10	13

```
eig = Eigenvalues[CΛΘX] / Apply[Plus, Eigenvalues[CΛΘX]]
```

{0.862531, 0.118987, 0.0184815}

The parameter Z:

```
z = eig[[1]] / (eig[[2]] + eig[[3]])
```

6.27439

Here are the eigenvectors, indeed approximately proportional to {1,0,0}, {0,1,0} and {0,0,1}:

```
Round[100 Map[# / Max[Abs[#]] &, Eigenvectors[CΛΘX]]] // MatrixForm
```

100	-24	-1
-24	-100	-15
-3	-14	100

```
SmoothHistogram[
 Transpose[ΛΘX],
 PlotRange → {{-2, 2}, All},
 Filling → 0,
 ImageSize → 500,
 PlotStyle → {{Black, Thick}, {Blue, Thick}, {Red, Thick}},
 PlotRangeClipping → True,
 Axes → None,
 Frame → True,
 FrameStyle → Table[{Black, Thin}, {4}],
 FrameTicksStyle → {Black, Thin},
 BaseStyle → Directive[FontFamily → "Times", FontSize → 24],
 PlotLabel → "ΛΘX histograms"
]
```

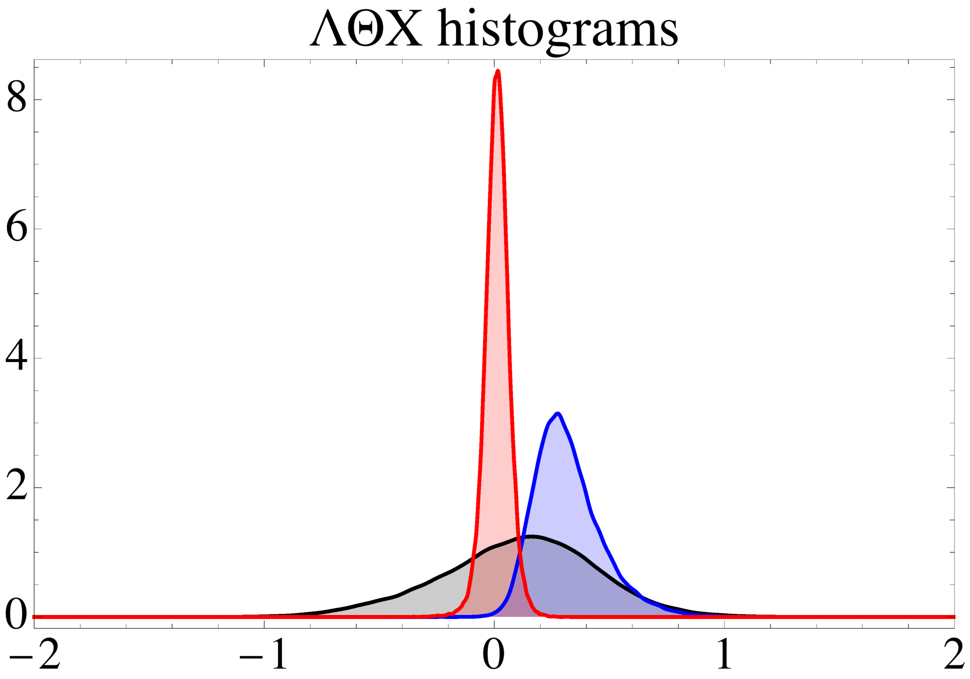

We find the means and standard deviation of Λ, Θ, X:

```
{{μΛ, σΛ}, {μΘ, σΘ}, {μX, σX}} =
 Map[{Mean[#], StandardDeviation[#]} &, Transpose[ΛΘX]];
Print["μΛ = ", μΛ, ", σΛ = ", σΛ];
Print["μΘ = ", μΘ, ", σΘ = ", σΘ];
Print["μX = ", μX, ", σX = ", σX];
```

```
μΛ = 0.102981, σΛ = 0.335204

μΘ = 0.320356, σΘ = 0.146483

μX = 0.0119556, σX = 0.0530376
```
