# Supplementary figures and images for: Colors of the Sublunar
Source: Iperception. 2017 Sep 29;8(5):2041669517733484. doi: 10.1177/2041669517733484 (PMC5624368; doi:10.1177/2041669517733484)

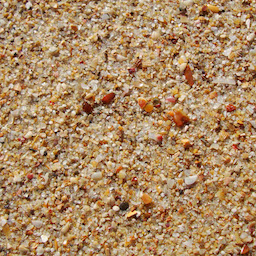

Supplement: Supplementary material [file Sand.jpg]
